# Multifaceted Role of Neuropilins in the Immune System: Potential Targets for Immunotherapy

**DOI:** 10.3389/fimmu.2017.01228

**Published:** 2017-10-10

**Authors:** Sohini Roy, Arup K. Bag, Rakesh K. Singh, James E. Talmadge, Surinder K. Batra, Kaustubh Datta

**Affiliations:** ^1^Department of Biochemistry and Molecular Biology, University of Nebraska Medical Center, Omaha, NE, United States; ^2^Department of Microbiology and Pathology, University of Nebraska Medical Center, Omaha, NE, United States

**Keywords:** neuropilin-1, neuropilin-2, immune cells, dendritic cells, macrophages, regulatory T cells, tolerance

## Abstract

Neuropilins (NRPs) are non-tyrosine kinase cell surface glycoproteins expressed in all vertebrates and widely conserved across species. The two isoforms, such as neuropilin-1 (NRP1) and neuropilin-2 (NRP2), mainly act as coreceptors for class III Semaphorins and for members of the vascular endothelial growth factor family of molecules and are widely known for their role in a wide array of physiological processes, such as cardiovascular, neuronal development and patterning, angiogenesis, lymphangiogenesis, as well as various clinical disorders. Intriguingly, additional roles for NRPs occur with myeloid and lymphoid cells, in normal physiological as well as different pathological conditions, including cancer, immunological disorders, and bone diseases. However, little is known concerning the molecular pathways that govern these functions. In addition, NRP1 expression has been characterized in different immune cellular phenotypes including macrophages, dendritic cells, and T cell subsets, especially regulatory T cell populations. By contrast, the functions of NRP2 in immune cells are less well known. In this review, we briefly summarize the genomic organization, structure, and binding partners of the NRPs and extensively discuss the recent advances in their role and function in different immune cell subsets and their clinical implications.

## Introduction

Neuropilins (NRPs) are multifunctional, single-pass transmembrane, non-tyrosine kinase surface glycoproteins that are expressed in all vertebrates with an important role in a wide range of physiological processes including development, axonal guidance, angiogenesis, immunity, as well as in pathological conditions such as cancer (1–9). They were originally identified based on their role in axonal guidance and neural development. Reports have demonstrated that NRPs mainly act as coreceptors for vascular endothelial growth factor (VEGF) and the class III Semaphorin family of molecules by interacting with VEGF receptors and Plexins, respectively. However, other ligands for NRPs have also been reported. The two isoforms, such as neuropilin-1 (NRP1) and neuropilin-2 (NRP2), are often upregulated in various clinical disorders, including cancer, where they increase the oncogenic activities of malignant cells by promoting survival, inducing angiogenesis and lymphangiogenesis and contribute to therapy resistance. NRP1 and NRP2 are expressed in various immune cells, such as macrophages, dendritic cells (DCs), T cells, B cells, and mast cells where they regulate a myriad of functions, including development, migration and recruitment, communication between different immune cells as well as regulation of immune response, under normal physiological condition and during pathological disorders. They are also detected in osteoclasts and osteoblasts where they regulate bone homeostasis. NRP1 is characterized mainly in T cell subsets, and to a lesser extent in macrophages and DCs. In comparison, NRP2 is less studied and poorly characterized. Despite growing evidences for immune regulatory functions by NRPs, knowledge of their ligands and pathways are minimal.

In this review, we summarize the genomic organization, different isoforms, and expression of the NRPs in normal physiology as well pathological conditions and what is known about their role and function in the different myeloid and lymphoid cells and osteoimmunology. Finally, we briefly review how we can develop NRP-based immunotherapies and their consequences.

## Genomic Organization, Protein Structure, and Splice Variants of NRP1 and NRP2

**Figure 1:**
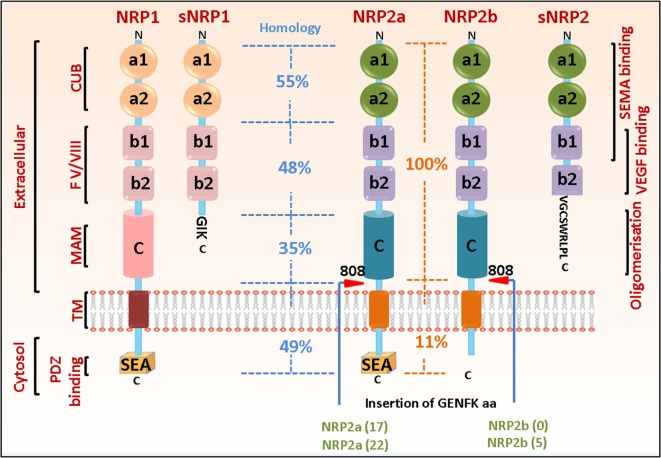
Neuropilin (NRP) domain structure and splice variants. The general domain structure of neuropilin-1 (NRP1) and neuropilin-2 (NRP2) is shown. There is an N-terminal extracellular domain for ligand binding, followed by a single-pass transmembrane domain and a short cytosolic tail. The extracellular domain comprises of two CUB, two b1/b2 and one MAM domain. The sites for binding different ligands are indicated. Both NRP1 and NRP2 can exist as multiple splice variants. Soluble isoforms (sNRP1 and sNRP2) contain truncated extracellular domain but lack the transmembrane and cytosolic regions and can act as decoy receptors to blunt NRP function. NRP2 can exist as two splice forms, NRP2a and NRP2b, which share only 11% homology in their C-terminus, therefore, being capable of regulating different signaling pathways. The percentage of sequence homology in the different extracellular and cytosolic domains of NRP1 and NRP2 as well as between NRP2a and NRP2b are indicated. The C-termini of both NRP1 and NRP2a contain a PDZ binding motif (SEA) that can act as docking site for interacting partners. Red arrowheads indicate insertion at residue 808 in NRP2 of five amino acids GENFK giving rise to different splice variants of NRP2a and NRP2b. The percentage amino acid homologies between the domains of full length NRP1 and NRP2 isoforms and between the NRP1 and NRP2a/NRP2b isoforms are indicated.

Neuropilins comprise of two homologous isoforms, such as NRP1 and NRP2, encoded by distinct genes on different chromosomes (10p12 for NRP1 and 2q34 for NRP2), that arose due to gene duplication and are structurally similar with overlapping sets of ligands and functions. Each gene contains 17 exons and 16 introns and similarly mapped exon–intron junctions (10). Both NRP1 and NRP2 exhibit similar domain structure, comprising an N-terminal extracellular domain followed by a transmembrane region and a short cytosolic tail of 43–44 amino acids. The extracellular domain contains two CUB (complement binding factors C1r/C1s, Uegf, bone morphogenetic protein 1) (a1/a2) domains, two factor V/VIII coagulation factor homology (b1/b2) domains, a b–c linker followed by a MAM (homologous to meprin protease, A5 antigen, receptor tyrosine phosphatase μ and К)(c) domain. The CUB domain is required for the binding of the Semaphorin group of ligands. The b1/b2 domains, characteristics of coagulation factors and of discoidin proteins, are known for binding with anionic phospholipids on the cell surface, thereby playing a role in cell–cell adhesion. This is the site where ligands such as Semaphorins and VEGFs interact. The MAM domain is important for homo- or heterodimerization of the receptors (Figure 1).

Since their discovery, multiple isoforms (splice variants) have been reported for both NRPs (Figure 1). For example, NRP1 can exist as either a membrane bound or soluble form. Interestingly, many of the isoforms display decoy functions to full size NRP1. For instance, Gagnon et al. and Rossignol et al. reported two soluble forms of NRP1, such as _s12_NRP1 and _s11_NRP1, due to pre-mRNA processing in intron 12 and intron 11, respectively (10). Since splicing occurs in the b–c linker region, the soluble isoforms contain a and b domains but lack the transmembrane and cytosolic residues. _S12_NRP1 acts as a decoy and inhibits VEGF_165_ binding to NRP1. Rat prostate carcinoma cells expressing recombinant _s12_NRP1 gave rise to tumors characterized by extensively hemorrhaged and damaged vessels and increased number of apoptotic tumor cells (11). Two additional soluble forms, such as sIIINRP1 and sIVNRP1, were detected in normal human as well as several tumor tissues. Recombinant sIIINRP1 and sIVNRP1 act as ligand trap, antagonized the effect of NRP1 and affected breast cancer cell migration (12). Another splice variant of NRP1 was recently identified (lacking a small sequence of seven amino acids, located two residues downstream of the *O*-glycosylation site and hence, less glycosylated) which when overexpressed in prostate cancer cells in nude mice, significantly reduced tumor burden and decreased tumor cell proliferation and migration (13). However, further studies are required to fully understand why different splice forms exist under different conditions, whether they arise as a host response under specific conditions, and the signaling pathways they govern.

Neuropilin-2 can also exist as either a membrane bound or a soluble form. Membrane bound NRP2 can exist as two splice variants, such as NRP2a and NRP2b, which differ only at the last 100 amino acids of their cytosolic tail. NRP2a exhibits 44% sequence homology with NRP1 at the amino acid level and may have overlapping functions. The extracellular domain of NRP2b is identical to that of NRP2a, but the transmembrane and cytosolic domains share only 11% homology. In humans, Rossignol et al. reported two splice variants of NRP2a, NRP2a(17), and NRP2a(22). NRP2a(17) results from the insertion of 17 amino acids after residue 809, located between the MAM and transmembrane domains, while NRP2a(22) has an additional 5 amino acids within the 17 amino acid residue in NRP2a(17) due to alternate splicing (10). NRP2b also exists as two splice variants, such as NRP2b(0) and NRP2b(5), resulting from alternate splicing between exon 15 and exon 16b and insertion of 0 or 5 amino acids after residue 808. NRP2b(0) was reported to be more abundantly expressed than NRP2b(5). As mentioned earlier, NRP2a and NRP2b have divergent C-termini, indicating they may bind different proteins and govern distinct molecular pathways (10). However, few studies have addressed this. Recently, a prometastatic role for NRP2b was reported in non-small cell lung carcinoma, whereas NRP2a had opposite effects in promoting metastasis and therapy resistance (14). However, additional studies are required to fully understand why these different splice forms are expressed in different tissues and their functions under different conditions. This is critical as potential therapies may be developed targeting specific splice variants for treating different clinical conditions. In mice, four splice variants of NRP2 have been reported so far due to alternative splicing, resulting in the insertion of 0, 5, 17, and 22 amino acids after residue 809. These variants may not have differential ligand binding properties; however, insertion of additional amino acids in the b–c linker transmembrane domains might alter their ability to homo- or hetero-dimerize with other receptors, thereby affecting distinct downstream signaling pathways. Interestingly, a soluble isoform for NRP2 (_s9_NRP2, 62.5 kDa) has been reported and contains the a1/a2 domain, the b1 domain but only portion of the b2 domain. Alternate splicing results in the inclusion of an intron in the b2 domain and the presence of an in-frame stop codon terminates the translation resulting in the soluble form that does not contain the last 48 amino acids in the b2 domain, the b–c linker, the transmembrane domain, and the cytosolic tail (10). Recently, a novel decoy function of s9NRP2 in sequestering VEGFC and inhibiting the oncogenic VEGFC/NRP2 signaling has been reported in prostate cancer cells where it significantly reduced the formation of prostatospheres (15). This opens the exciting possibility of using _s9_NRP2 as a therapeutic strategy in treating tumors heavily relying on the VEGFC/NRP2 axis for their survival.

## Ligands for NRP1 and NRP2

A wide variety of ligands have been reported for NRP1 and NRP2. NRPs are well known for binding Class III Semaphorins and selected members of the VEGF family, two structurally unrelated classes of ligands with different biological functions. Semaphorins comprise a large family of proteins and are categorized into seven different classes. They trigger signaling by binding with Plexins on the cell surface and have been associated with various functions in developmental biology, normal physiological processes, immunity, as well as pathological conditions. Class III Semaphorins are secreted molecules that bind to the a1/a2/b1 domain of NRP1 or NRP2 with different affinities and specificities and form a holoreceptor complex with NRPs and PlexinA1 or PlexinA2. For instance, the major ligand for NRP1 is Semaphorin 3A (Sema3A); although, it can also bind with other Class III Semaphorins, such as Sema3F, albeit with lower affinity. NRP2 mainly binds to Sema3C and 3F, but not 3A. NRPs also bind different isoforms of several VEGF family members, with different specificities and functional consequences. Interestingly, NRPs can also bind other growth factors; however, in most cases, they are not indispensable as coreceptors and only enhance the signal. For example, NRPs can bind with transforming growth factor beta 1 (TGF-β1) and signal through the canonical Smad2/3 pathway exerting antiapoptotic and antiproliferative effects. They are also reported to bind to c-Met and platelet-derived growth factor and are important for tumor progression. Recently, NRP1 has been reported to act as a receptor for extracellular microRNAs (miRNAs). miRNAs are common in biological fluids and circulate either in encapsulated form or bound to protein argonaute-2 (AGO2). NRP1 binds AGO2/miRNA complexes and facilitates their cellular internalization. Interestingly, VEGF was not found to compete with miRNAs for binding to NRP1 (16). It is now well accepted that miRNAs can mediate a wide array of functions at distant locations, both under normal as well as pathological conditions. They have been associated with tumor progression, epithelial to mesenchymal transformation, and metastasis as well as disease prognosis (17, 18). Also, NRP1 is overexpressed in various disorders including malignancies (19–29). That NRP1 acts as a natural receptor for AGO2/miRNA complex may have important consequences under normal and pathophysiological conditions.

## Posttranslational Modification of NRPs

There are several reports, which indicate that both the NRPs undergo posttranslational modifications. NRP1 is often modified by the covalent addition of glycosaminoglycan (GAG) chains covalently attached to a single conserved Ser^612^ residue in the b–c linker region of the receptor (30). Structurally, GAGs are a repeating disaccharide unit containing *N*-acetylglucosamine (GlcNAc) or *N*-acetylgalactosamine (GalNAc) and a uronic acid (glucuronate or iduronate) or galactose. Depending on the structure of the core disaccharide, GAGs can either be heparan sulfate (HS-GAG), chondroitin sulfate (CS-GAG), keratin sulfate, or hyaluronic acid. Frankel et al. reported the existence of two populations of NRP1 in several human tumor cell lines. One fraction consists of the *N*-glycosylated protein, and the other is posttranslationally modified by the addition of CS-GAG. Surprisingly, CS-GAG modification inversely correlated with tumor cell invasiveness (31). CS-GAG NRP1 is predominantly expressed in the vascular smooth muscle cells. In HUVEC cells, NRP1 expressed equivalent amount of CS-GAG and HS-GAG. GAG modification on NRP1 enhances its binding to VEGF; however, CS-GAG NRP1 may act as a decoy receptor under certain circumstances. On the other hand, HS-GAG NRP1 may bind multiple NRP1 molecules and promote NRP1 clustering. Such a cluster can bind VEGFR2 in presence of VEGF and stabilize the complex, prevent internalization of VEGFR2, resulting in enhanced signaling (30). By contrast, there are no reports for such modification on NRP2. In DCs, NRP2 undergoes posttranslational modification by the addition of polysialic acid (PSA) chains to mucin-type *O*-linked glycans between the b2 and c domains (32, 33). NRP2 polysialylation regulates CCL21-driven trafficking of DCs to the secondary lymphoid organs and modulates interactions between DCs and T lymphocytes (34). NRP1 has also been recently reported to undergo polysialylation at a level 50% of that of NRP2, although the exact biological implication of this remains unknown (35). These functions of NRPs will be discussed in more details in the following sections.

## Phenotype of Genetically Engineered Mouse Models for NRP1 and NRP2

Depending on the genetic background of the mice, NRP1 depletion can be embryonic lethal at E10.5–13.5. The embryos exhibit severe defects in cardiac and vascular development and disorganized nerve fiber projections (36–38). Transgenic mice with NRP1 overexpression also die *in utero* at E12.5 and exhibited excess capillary formation, extensive hemorrhage, and defects in the nervous system (39). Mice with endothelial specific depletion of NRP1 also show embryonic mortality accompanied with multiple defects in the cardiac and vascular development (40–42). VEGFA is indispensable for vascular development and exerts its functions through interaction with its receptors VEGFR1/2 and NRP1. The cytosolic tail of NRP1 has a PDZ binding motif where it can interact with a protein named GIPC1. The latter is important for arterial morphogenesis and signals through VEGFR2. A knockin transgenic mice, where NRP1 lacked the cytosolic domain, exhibited impaired arterial morphogenesis and reduced body size (43, 44). This defect was attributed to impaired trafficking of endocytosed VEGFR2 from Rab5^+^ to EEA1^+^ endosomes in absence of interaction between NRP1 and GIPC1. This resulted in PTPN1 (PTP1b)-mediated dephosphorylation of VEGFR2 at Y1175 and deregulated arteriogenic ERK signaling.

Neuropilin-2 knockout mice are viable, proceed to adulthood but show reduction in smaller lymphatic vessels and impaired development of cranial nerves, spinal sensory axons and defects in the arrangement of fiber tracts in the adult brain (45–47). Interestingly, these mice exhibited lower bone mass, which could be attributed to an increased number of osteoclasts and/or a reduced number of osteoblasts (48). This suggests that NRP2 has a role in normal bone homeostasis, which is particularly important in cancer patients where tumor metastasis to bone can result in deregulation of normal homeostasis process. That NRP2 clearly has a role in maintaining normal bone health may provide a target for the treatment of cancers that metastasize to bone. Depletion of both NRP1 and NRP2 was lethal at E8.5 resulting in severe defects in vasculature development, marked by the presence of large avascular areas in the yolk sac and gaps between blood vessel sprouts (49). Mice deficient for NRP1 but heterozygous for NRP2 or *vice versa* were also embryonically lethal at E10–10.5. These mice exhibited severe defects in vasculature and their yolk sacs failed to develop branching arteries and veins and a capillary bed and exhibited extensive avascular spaces between the blood vessels. Overall, these reports identify a crucial role for NRPs in cardiovascular and neuronal development as well as maintenance of bone homeostasis under physiological conditions.

## Role and Function of NRP1 and NRP2 in the Immune Cells

The immune system comprised of two compartments, such as the innate and adaptive systems. The innate immune system mainly comprise of cells of myeloid lineage, macrophages, DC, neutrophils, eosinophils, basophils, and natural killer (NK) cells, whereas the adaptive arm includes T and B cells. A complex interplay occurs between the immune cells and is crucial for controlling infectious diseases and neoplasia. Studies in recent years have shown that NRPs are expressed in various subsets of immune cells and are important for regulating immune response. In the following sections, we will briefly review what is known about the role of NRPs in various immune cells under normal and pathophysiological conditions.

In recent years, NRP1 and NRP2 have been shown to be expressed on DCs, macrophages, T cell subpopulations, and mast cells and to be crucial for regulating immune responses, under normal as well clinical conditions. These have been summarized in Table 1. For example, NRP1 is involved in the formation of immunologic synapse between DCs and naïve T cells (50). The expression of NRP1 has also been reported in immature thymocytes (51). Interestingly, NRP1 expressed on the surface of DCs can be transferred to T cells by the process of trogocytosis a suggestion supported by the observation that T cells start expressing NRP1 within 15 min of coculture with DCs. NRP1 is also considered to be a marker for murine T_reg_s where its expression correlated with immunosuppression (52, 53). By contrast, its expression on and use as a marker for human T_reg_s is still under debate and is proposed to be able to distinguish between thymic-derived and mucosa-generated peripherally derived T_reg_ cells (54, 55). NRP1 is also selectively expressed on a subset of T follicular helper (Tfh) cells in secondary lymphoid organs in humans and correlates with B cell differentiation (56). Recently, Milpied et al. reported NRP1 expression in recent thymic emigrant natural killer T (NKT) cells but not on mature NKTs (57, 58). NRP2 expression in macrophages, DCs, and T cells is endowed with complex functions such as tissue homeostasis, migration, and immune modulation. under normal as well as pathological and clinical conditions. These will be discussed in greater details in the relevant sections below.

**Table 1:** Expression and functions NRPs in the immune system and related diseases.

**NRPs**	**Expression**		**Functions**	**Related diseases**	**Reference**
	**Immune cells**	**Cellular context**			

NRP1	Dendritic cells (DCs) Plasmacytoid DC		Role in production of IFN-α (?) and viral clearance (?) Increases susceptibility to HTLV-1 virus Migration and induction of immunosuppression in TIDCs, correlation with disease progression (?)	Viral infection Retroviral infection Cancer	(82) (88) (106)
	
	myeloid DC (mDC)		Formation of immunological synapse (IS) with T cells by homophilic interaction between NRP1 on DC and T cells Induction of immune tolerance and prevent aberrant activation of T cells Reorganization of actin cytoskeleton and transmigration of DC to lymph node through Sema3A/NRP1/PlexinA1 axis Increases susceptibility toward HTLV-1 virus		(50) (50, 69, 77) (84) (87–89, 91, 92)
	
	Macrophages, microglia		Developmental vascularization Promotion of M2 type polarization, phagocytosis, induction of T_reg_s, and immunosuppression Migration to the hypoxic core of solid tumors through Sema3A/NRP1/PlexinA1/PlexinA4 axis Protumorigenic activities of TAMs Negative regulation of TAM proliferation in certain tumors through Sema3A binding (?)	EAE Cancer Cancer Cancer	(44, 123–125) (147, 148) (133, 134) (133, 134, 136, 137, 139) (135)
	
	Osteoclasts Osteoblasts		Osteoprotection by preventing osteoclastogenesis and promoting formation of osteoblasts		(156)
	
	T cells Thymocytes (premature)		Adhesion and deadhesion to thymic epithelial cells Actin reorganization (?) Egress from thymus (?)		(51, 179)
	
	T_reg_		Immunosuppression and induction of tolerance Migration of T_reg_ to the tumor microenvironment Stability and function of T_reg_s	EAE GVHD Cancer	(181, 186–191, 200) (209) (218)
	
	CD8^+^ effector and memory cells	Viral infection Priming by liver sinusoidal endothelial cells	Not known Not known		(231) (233)
	
	Mucosal CD8^+^Foxp3^+^ cells Tumor-infiltrating CD8^+^ cells	Exposure to gut specific antigen	Suppressed CD4^+^ T cell proliferation *in vitro* Not known	Metastatic melanoma	(232) (234)
	
	NKT, recent thymic emigrant IL-17-producing iNKT cells		Role in development (?) Egress from thymus (?) Interaction between iNKT and macrophages (?)		(57, 58)
	
	Tfh		B cell differentiation Migration to and retention of Tfh in secondary germinal center of lymphoid organs	Angioimmunoblastic T cell lymphoma (AITL)	(56)
	
	Basophils		Not known		(264–266)
	
	Mast cells		Not known		(264–266)

NRP2	DCs mDCs		Interaction with T cells Chemokine guided migration		(33) (34, 110)
	
	Macrophages/microglia Alveolar, bronchial, and intravascular macrophages		Not known		(136)
	
	Microglia	LPS challenge	Negative feedback regulation of proinflammatory responses		(154, 155)

	Peritoneum macrophage		Phagocytosis (?)		(153)
	
	TAMs		Not known		(136, 151)
	
	Osteoclasts/osteoblasts		Negative regulation of osteoclast number Promotion of osteoblast formation	Osteosarcoma	(48, 161–163)
	
	T cells CD4^+^CD8^+^ developing thymocytes		Migration Differentiation (?)		(249)
	
	VY9Vδ2 T cells		Not known	T-ALL T-LBL Non-Hodgkin’s lymphoma	(260)
	
	CD4^+^ effector T cells		Negative regulation of proliferation Immunomodulation in graft transplant	GVHD	Published abstract, ATC, 2015
	
	CD4^+^Foxp3^+^ T_reg_		Negative regulation of proliferation Immunomodulation in graft transplant	GVHD	Published abstract, ATC, 2015
	
	Basophils		Not known		(264–266)
	
	Mast cells		Not known		(264–266)

*NRPs, neuropilins; NRP1, neuropilin-1; NRP2, neuropilin-2; Sema3A, Semaphorin 3A; Tfh, T follicular helper; HTLV, human T-cell leukemia virus; TIDCs, tumor-infiltrating DCs; TAM, tumor-associated macrophage; EAE, experimental autoimmune encephalomyelitis; NKT, natural killer T; T-ALL, T-cell acute lymphoblastic leukemia; T-LBL, T-cell lymphoblastic lymphoma; iNKT, invariant natural killer T; GVHD, graft-versus-host disease; T_reg_, regulatory T cell*.

## NRP1 and NRP2 in DCs

Neuropilins have been implicated in different aspects of DC biology. DCs are specialized antigen-presenting cells (APCs). Following processing and presentation they can prime a T cell response, thus, bridging the innate and adaptive immune responses. They are also crucial for the maintenance of tolerance under steady state conditions. Over the years, studies have shown that DCs form a heterogeneous population, with unique as well as overlapping functions. They are two broad types, such as conventional or myeloid DCs (cDC or mDC) and plasmacytoid DCs (pDCs). Depending on the cellular context and cues they receive from the tissue microenvironment, both subtypes can prime immunogenic as well as tolerogenic responses. There are several reviews that have summarized the phenotypic and functional diversity of DC subsets (59–67). Briefly, both cDCs and pDCs differentiate from a common myeloid progenitor in the bone marrow; however, a fraction of pDCs originate from the common lymphoid progenitor. cDCs are specialized in antigen presentation *via* both MHC-I and MHC-II pathways and stimulate T cell responses to control intracellular and extracellular pathogens. pDCs comprise less than 0.2–0.8% of peripheral blood cells in human and reside in secondary lymphoid organs during steady state conditions (68). They are the primary source of type I interferons (α/β) following viral infection and ligation of toll-like receptor (TLR) 7 and TLR9. The secreted IFN-α can activate cytotoxic T cell responses and elimination of virus-infected cells or can hyperactivate and eventually deplete T cells through chronic immune activation/exhaustion and result in progression of viral infections (65). Due to DC expression of MHC class II molecules and costimulatory molecules such as, CD40, CD80, and CD86, pDCs can present antigens to CD4^+^ T cells, albeit less efficiently than cDCs. Depending on the context, pDCs can also induce a tolerogenic response by favoring the formation of T_reg_s, inhibiting the formation of and inducing apoptosis of T effector cell populations. Interestingly, during inflammation, circulating monocytes can also differentiate to become DCs, known as monocyte-derived DC (moDC), to provide an emergency backup; these moDCs are characterized by the expression of MHC-II and production of TNF-α, nitric oxide (NO), and IL-12 for priming effector T_H_1 and cytotoxic T cell responses. In this review, we will mainly focus on what is known about NRPs and their functions in the different DC subsets.

**Figure 2:**
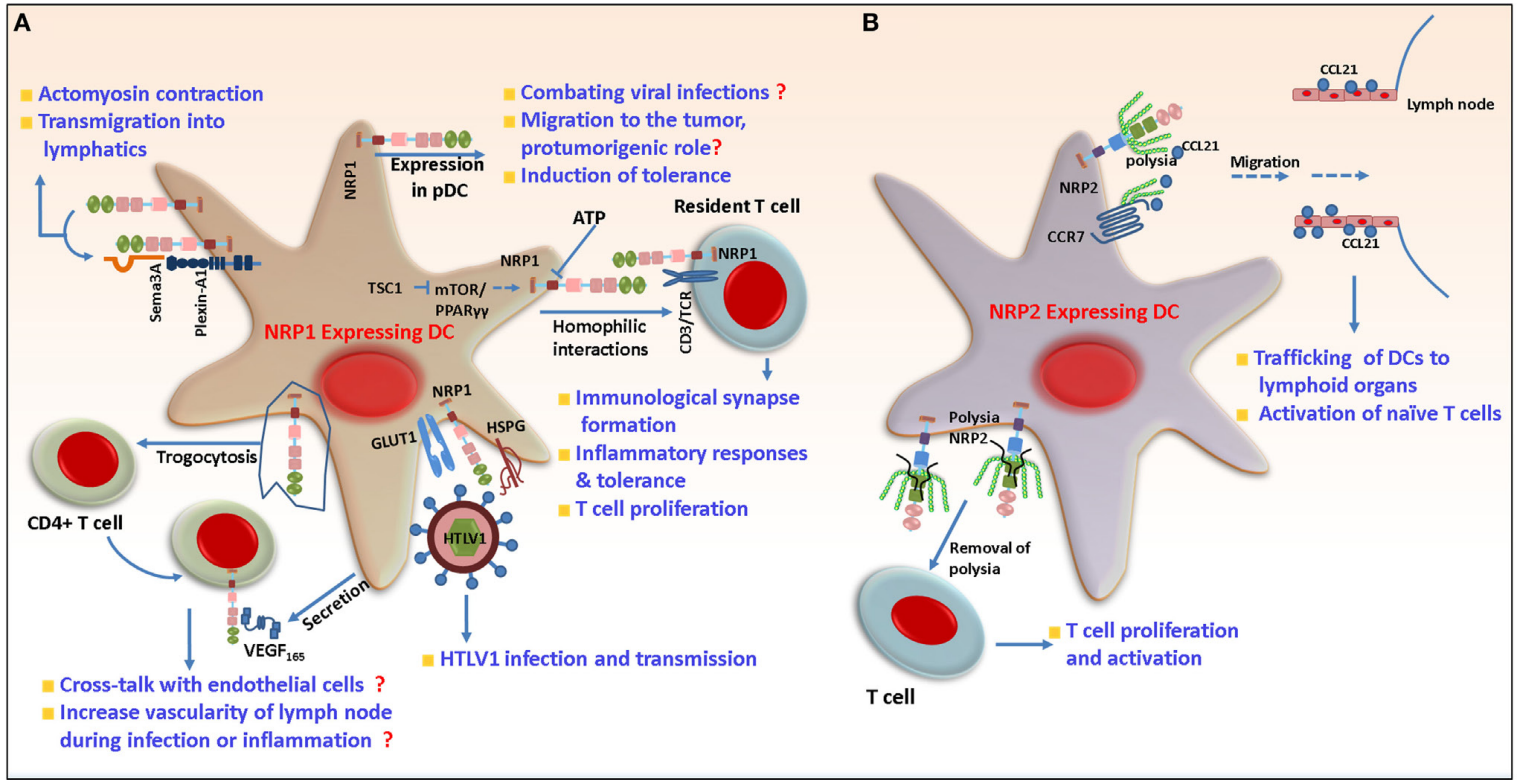
Role of neuropilin-1 (NRP1) and neuropilin-2 (NRP2) in dendritic cells (DCs). **(A)** Following antigen exposure, DCs need to migrate to lymphoid tissues to activate T cells. NRP1 in concert with PlexinA1 and Semaphorin 3A (Sema3A) regulates cytoskeleton rearrangement in DCs and their transmigration to lymphatics. NRP1 is involved in the formation of primary immune synapse with T cells and positively regulates their proliferation. In addition to this, NRP1 can be transferred from DCs to T cells by trogocytosis and then be carried and presented *in trans* to endothelial cells, for increasing LN vascularity during inflammation. NRP1 increases the susceptibility of DCs to human T-cell leukemia virus-1 infection by acting as a receptor for the virus on the cell surface and mediates virus transmission to non-infected cells. During viral infection, it regulates the production of IFN-α through unknown mechanism. NRP1 is also important for migration of DCs to tumor microenvironment and induction of immunosuppression. **(B)** NRP2 is polysialylated and mediates migration of DCs in response to CCL21 to lymph nodes. The polysia may acts as a protective shield to prevent inappropriate interaction of NRP2 with other molecules until it reaches LNs and is then shed. Non-polysialylated NRP2 then interacts with T cells and regulates their proliferation.

As mentioned earlier, the functional activity of DCs depends on the cues derived from the tissue microenvironment. Immature DCs (iDCs) are recruited to the inflamed site where, following exposure to antigen, they undergo maturation, migrate to the lymphoid organs to prime naïve T cells and thus initiate the primary immune response. NRP1 protein was detected in mDCs and resting T cells isolated from human peripheral blood. During the formation of immunological synapses (ISs) between DCs and allogenic T lymphocytes, NRP1 promoted cell–cell adhesion *via* homophilic interactions and colocalized with CD3 at the contact zone, indicating a potential role for NRP1 in the initiation of primary immune response (Figure 2). Indeed, T cell proliferation is diminished upon treatment of either DCs or resting T cells with NRP1 blocking antibody before the formation of immune synapse (50). Sema3A secreted from activated DC and T cell can also bind to NRP1 on T cells and inhibit T cell proliferation by inhibiting actin cytoskeleton reorganization. This prevented the early events necessary for T cell activation (69). In contrast to their immunostimulatory function, exposure of iDCs to self-antigens derived from apoptotic cells during tissue homeostasis induces immune tolerance. This is characterized by enhanced secretion of immunosuppressive cytokines such as IL-10 and inhibition of IL-12 expression, which suppresses effector T cell responses, facilitates T cell anergy and the differentiation of T_reg_ cells and is indispensable for maintaining a tolerogenic response (70–76). In one study, Bles et al. demonstrated that treatment of mDCs with ATP-γs [a non-hydrolyzable analog of adenosine triphosphate (ATP)] significantly downregulated NRP1 expression (77). ATP has complex and multifaceted roles in immunity, as it is secreted from cells undergoing necrosis and serves as a key mediator of phagocyte recruitment. Release of ATP into the extracellular milieu can either trigger an immune response *via* formation of NALP3–ASC inflammasomes or induce tolerance, depending on the concentration of ATP and how quickly it gets hydrolyzed to immunosuppressive adenosine by membrane ectonucleotidases. Chronic exposure to low dose ATP induces a distorted maturation phenotype in DC with a decreased ability to secrete proinflammatory cytokines such as TNF-α and IL-12 and impaired T_H_1 responses (78). ATP-mediated downregulation of NRP1 in DCs indicates a probable role of this protein in DC-mediated inflammatory responses and tolerance induction. According to a recent study, NRP1 expression on DC may be regulated by the tuberous sclerosis complex I (TSCI); DC specific deletion of TSCI activates the mTOR/PPARγ pathway and upregulates NRP1 expression. This results in hyperproliferation and aberrant activation of naïve T cells in absence of antigen, indicating a requirement for NRP1 in maintaining naïve T cell quiescence under steady state conditions (79). NRP1 is also expressed on pDCs in the peripheral blood, bone marrow, and cord blood (50, 80, 81). Blocking of NRP1 with anti-NRP1 reduces the production of IFN-α by pDCs, although the exact underlying mechanism is unclear. Since interferons are crucial for successfully combating viral infections, it has been hypothesized that NRP1-mediated IFN-α secretion in pDCs may affect virus clearance *in vivo* (82). NRP1 expressed on mDCs can be transferred to activated as well as non-activated CD4^+^ T cells by trogocytosis, an active process to mediate transfer of membrane bound molecules between different immune cell components (83). This transferred NRP1 could then bind VEGF_165_ secreted into the surrounding milieu by DCs. The physiological significance of this requires further study; however, it is tempting to speculate that T cells transport VEGF_165_ through the circulation and during inflammation, present it *via* cross talk to endothelial cells for their activation. Following exposure to antigen, DCs traffic from peripheral tissue and transmigrate through the endothelium *via* lymphatics to secondary lymphoid organs and prime T cells. NRP1 signals through PlexinA1 and Sema3A and is important for the migration of murine DCs to lymphoid organs. Sema3A secreted from the lymphatics binds PlexinA1/NRP1 at the edge of DCs, resulting in myosin light chain phosphorylation, actomyosin contraction, and remodeling of DC cytoskeleton, thereby promoting DC transmigration (84) (Figure 2). Recently, it has been shown that Class III Semaphorins (Sema3A, Sema3C, and Sema3F) induce F-actin reorganization in human mDCs. Interestingly, the authors observed that the Sema receptors—NRP1 and NRP2—have differential expression patterns on immature and mature DCs. While both receptors are expressed on iDCs, NRP1 is significantly reduced whereas NRP2 increased as the cells matured. This study reports that although equal amount of each of the Semaphorins bind to iDCs, relatively less Sema3A and more of Sema3C and Sema3F bind to mature DCs. This is in agreement with the divergent expression of the receptors on mature DCs. Sema3C bound partly and 3F bound predominantly through NRP2 and this binding is inhibited by antibody to NRP2 suggesting its involvement in DC migration. Thus, both NRP1 and NRP2 function through Class III Semaphorins, can influence human DC migration and thereby affect immune response (85). Further additional insight is needed into the functional consequences of differential expression of NRP1 and NRP2 in iDCs and mature DCs.

In a recent study, mDCs were shown to be more susceptible to human T-cell leukemia virus (HTLV) type I infection than their autologous T lymphocytes counterpart. HTLV is a retrovirus that can cause adult T-cell leukemia/lymphoma and a progressive neurological disease called HTLV-1-associated myelopathy/tropical spastic paraparesis. This eventually leads to blood–brain barrier breakdown and deregulation of the central nervous system (CNS) (86). Like many viruses, HTLV-1 infects and hijacks the cellular machinery to further infect T cells. The increased susceptibility of DC toward HTLV-1 correlated with increased NRP1 expression on the DCs, the latter being important for DC-T cell transmission of the virus (87, 88). Both mDC and pDC can be efficiently infected by the virus. NRP1 promotes the binding of HTLV-1 to the surface of DCs by physically interacting with the HTLV-1 envelope proteins (89). HTLV-1, which is transmitted through a viral synapse, enters non-transfected target cells *via* interaction with the GLUT-1 receptor (90). Interestingly, NRP1 colocalized with GLUT-1 at cell contact sites between the infected and non-infected cells, indicating these two molecules work in concert to mediate the fusion of viral and cell membrane and facilitate the transmission of HTLV-1 (89, 91, 92) (Figure 2). In another study, using myeloid-derived DCs, Lambert et al. demonstrated that HTLV-1 envelope surface subunit interacts with heparan sulfate proteogylcans (HSPG) *via* its C-terminal domain and attach to cell surface. The former also encodes a motif mimicking VEGF_165_ to bind to NRP1-b domain. The stable interaction between HTLV-1, HSPG, and NRP1 causes a conformational change in the envelope protein that facilitates its binding with GLUT-1 and fusion with cell membrane. Treatment with exogenous VEGF_165_ reduced susceptibility to HTLV-1 infection. This indicates a possible role of VEGF_165_ as a potent inhibitor of HTLV-1 infection (93). However, further studies are required to determine if these molecules are enough to explain HTLV-1 entry into cells and can be therapeutically targeted for treating patients infected with this virus in a clinical setting.

Work over the past decade has made it increasingly clear that immune cells comprise a substantial population in the tumor microenvironment (TME) and correlate with clinical outcome. Once in the TME, infiltrating immune cells are usually hijacked by tumor cells and rendered dysfunctional by tumor cell-derived factors that interfere with their normal function, thereby resulting in a loss of their immunostimulatory properties and immune evasion of tumor. DCs infiltrate solid tumors and are often associated with poor clinical outcome. These tumor-infiltrating DCs (TIDCs) are polarized toward a tolerogenic phenotype and can promote T_reg_ proliferation, resulting in T cell anergy and immune suppression (94–100). For example, pDCs infiltration of tumors is associated with a poor outcome measured as time to disease progression and overall survival. Further, in the TME, the ability of these tumor infiltrating pDCs to produce inflammatory cytokines and activate T cells is blunted. By contrast, they secrete transforming growth factor beta (TGF-β) and IL-10, promoting the formation of T_reg_s and the suppression of T cell proliferation. Intratumoral depletion of pDCs can stimulate an antitumor T-cell response, reduce tumor burden, and prevent metastasis in animal models (66, 101–105). NRP1 has been detected in pDCs isolated from patients with chronic lymphocytic leukemia (CLL) (106). NRP1 expression on pDCs could be involved in tumor trafficking of pDCs or tolerance induction. Confirmed insight into the function and prognostic value of NRP1 expression on tumor-associated DCs is still lacking, including the regulation of NRP1 expression by TIDCs. VEGF has been repeatedly reported to have an inhibitory role on DC function and maturation (107, 108). For example, VEGF inhibits DC maturation through blockade of NF-κβ pathway. Thus, when bone marrow-derived DCs are challenged with LPS in presence of VEGF, the latter, in concert with its coreceptor NRP1, impairs DC maturation. This is characterized by the downregulation of MHC-II and other costimulatory molecules, as well as, the expression of proinflammatory cytokines such as IL-12, TNF-α, IL-1β, and IL-6 (109). Observations of this nature are clinically relevant for the design of more efficient DC vaccines and the reprogramming of DC maturation in conditions where VEGF is present in the surrounding milieu. Although more detailed studies are needed to address how VEGF, in concert with NRP1, may influence the maturation and functional phenotype of different DC subsets in various pathological conditions, it is tempting to speculate that DC vaccines might be engineered to silence NRP1. This may facilitate TLR4 driven DC activation and T_H_1 immune responses.

In contrast to NRP1, the immunological analyses of NRP2 in different DC subsets are immature. NRP2 is expressed in human mDCs during maturation and its expression is upregulated by LPS. NRP2 is posttranslationally modified by the addition of PSA to a cluster of mucin-type *O*-linked glycans. The *O*-linked glycans are attached to four threonine residues located in a short stretch of 17 amino acids in the b–c linker domain. The polysialylation is mediated by the enzyme polysialyltransferase ST8SiaIV, one of the two mammalian polysialyltransferases (33). Polysialylated NRP2 negatively regulates the allo-interaction between DCs and T lymphocytes, such that removing polysia or treating the cells with anti NRP2 (which reduces NRP2-VEGF binding) results in increased DC-mediated T cell proliferation and activation. It has been suggested that polysialylation on NRP2 protects against interactions with other molecules until DCs traffic to LNs and activate T cells. In a recent report, it was reported that both membrane bound and soluble NRP2 were polysialylated. The MAM domain and *O*-glycan containing linker region are required, as well as sufficient, for polysialylation of NRP2 (35). In this study, it is suggested that ST8SiaIV recognizes and docks on the acidic surface of the MAM domain and polysialylates NRP2. The addition of PSA to a protein is a rare posttranslational modification and plays an important role in developmental biology, immune responses, as well as clinical disorders such as cancer. Polysialylated NRP2 regulates DC migration in response to the chemokine CCL21 by facilitating its binding to CCR7 on DCs (Figure 2). CCL21 is a critical lymph node chemokine, which when binds to its receptor CCR7, provides the cue for the induction of signaling cascades, such as JNK and Akt pathways, that eventually traffic DCs to lymphoid organs for the activation of naïve T cells (110). Interestingly, CCL21 has an extended basic C-terminus, which can bind to negatively charged polysia. Whether the polysia residues on NRP2 interact and bind to CCL21 increasing its availability to CCR7 is not clear requiring additional study. Indeed, Kiermaier et al. reported that in ST8SiaIV null mice, DCs were refractory to CCL21 and migrated less to the peripheral lymph nodes (pLNs) following inflammatory insult resulting in perturbed LN homeostasis. Given previous findings, one might attribute this defect to loss of polysialylation on NRP2 in ST8SiaIV^−/−^ mice. However, in contrast to earlier reports, using both *in vitro* and *in vivo* approaches, the authors reported that NRP2^−/−^ DCs did not have a migratory defect in response to CCL21. Their study indicates that polysialylated NRP2 is dispensable for murine DC migration and included evidence suggesting that apart from NRP2, CCR7 itself is polysialylated in DC and that binding of CCL21 to polysia on its receptor releases the ligand from its autoinhibitory inactive state thereby facilitating chemotaxis (111). In summary, polysia on CCR7 may compensate for NRP2 abrogation and facilitate DC chemotaxis in response to CC21. NRP2 can exist as two isoforms, such as NRP2a and 2b. Both isoforms are detected on mDC and target for polysialylation and regulate CCL21-driven chemotaxis with similar efficiency. However, the ratio of each isoform varies by donor (34), and extensive studies are required to understand if the isoforms have redundant or distinct functions in DCs and molecular pathways that are governed by each.

Overall, these reports suggest that both NRP1 and NRP2 play important roles in various aspects of DC biology, both in normal as well as pathological conditions.

## Function of NRPs in Macrophages

Macrophages are a heterogeneous and plastic hematopoietic cell and present in most tissues, acting as a bridge between innate and adaptive immunity. They can originate either from the yolk sac or the bone marrow and increasing number of studies have focused on their broad array of house-keeping functions. Majority of the tissues in our body harbor resident macrophages. They are highly plastic, extremely heterogeneous and undertake an array of house-keeping functions that range from clearance of cellular debris arising from regular turnover in tissues, iron homeostasis, immune surveillance, as well as response to and resolution of inflammation and facilitation of wound healing. Briefly, they originate from the erythro-myeloid progenitors in the yolk sac at embryonic day (E) 8.5 and are rarely replaced in the adult tissue. During any inflammatory response, however, circulating monocytes from the peripheral blood are recruited to the inflamed tissue to replace or aid the resident tissue macrophages. The latter act as sentinels for immune surveillance and are crucial for clearing apoptotic cell debris and cross presentation of self-antigens to T cells for the maintenance of homeostasis and tolerance (112–122). NRP1 has been reported on tissue-resident macrophages. For instance, TIE2^+^NRP1^+^ yolk sac-derived microglia/macrophages comprise a substantial population of tissue-resident macrophages during brain vascularization. They are also detected in proximity to endothelial tip cells and act as cellular chaperones for vessel fusion. However, selective depletion of NRP1 on macrophages/microglia was dispensable for normal vessel growth in the brain (44, 123, 124). In a recent study, Dejda et al. reported that NRP1^+^ macrophages/microglia were detected at sites of vessel anastomosis during retinal development but were dispensable for normal retinal angiogenesis (125). In human uterus, decidual macrophages have been reported to exhibit an immune suppressed phenotype that is crucial for the maintenance of the semi-allogenic fetus. Among these macrophages, those which were CD14^high^, CD11c^low^, and expressed NRP1, exhibited gene signature associated with extracellular matrix formation and tissue growth. Therefore, NRP1 expression in decidual macrophages may be important in the maintenance and growth of the uterine muscle cells during placental construction (126, 127).

**Figure 3:**
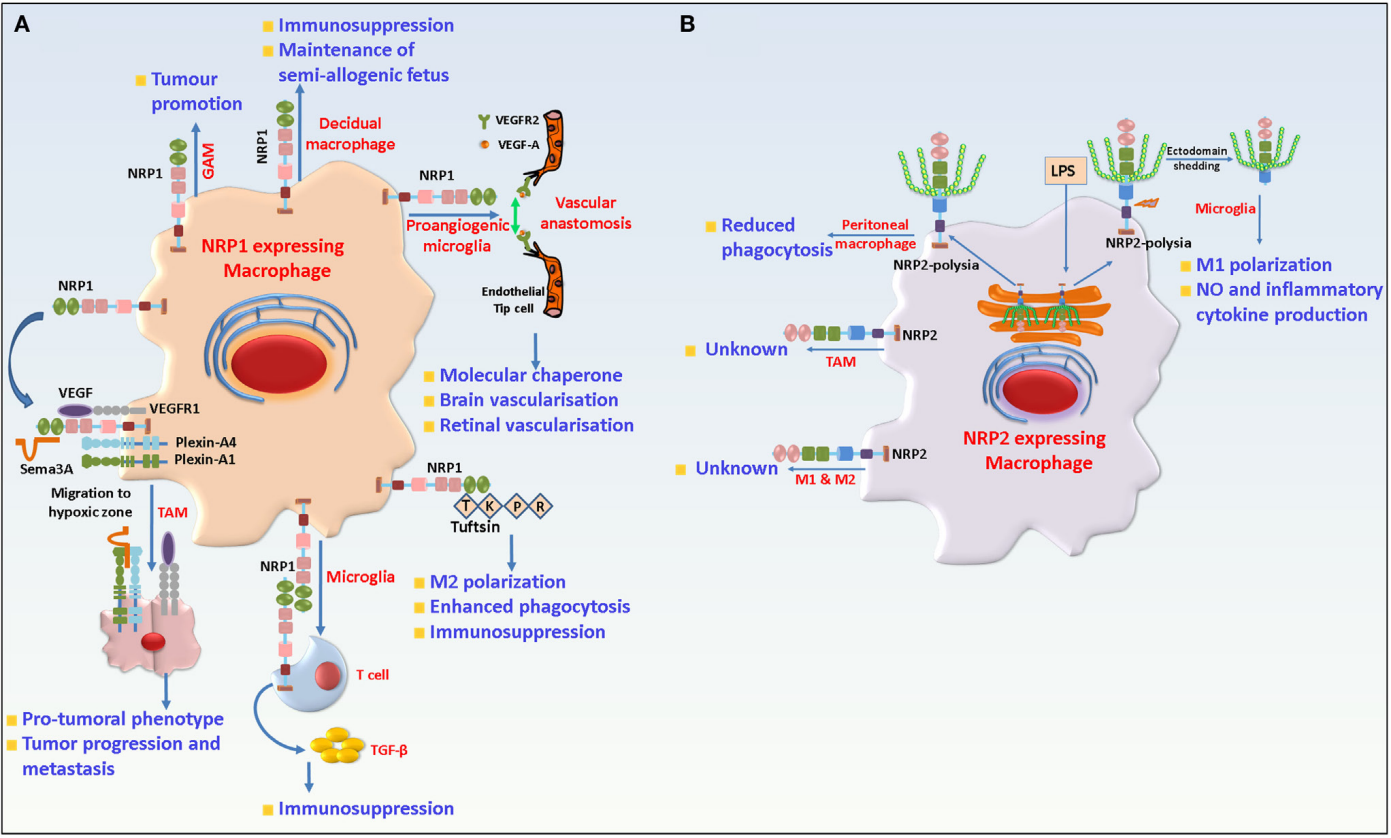
Role of neuropilin-1 (NRP1) and neuropilin-2 (NRP2) in macrophages and microglia. **(A)** NRP1 is detected in resident macrophages where it is involved in developmental vasculogenesis and maintenance of fetus. In TAMs, NRP1 in concert with PlexinA1/PlexinA4, binds Semaphorin 3A (Sema3A) and is responsible for migration of the former to the hypoxic core of the tumor. Once in the hypoxic core, NRP1 is downregulated and hence TAMs lose their responsiveness to Sema3A and remains trapped there and favor tumor growth by promoting angiogenesis and immunosuppression. In microglia, NRP1 promotes M2 polarization and phagocytosis of cellular debris and is involved in interaction with regulatory T cell to trigger transforming growth factor beta (TGF-β) release and immunosuppression. In addition, NRP1 also plays a protumorigenic role in GAMs. **(B)** NRP2 is expressed in microglia, tissue-resident (M2) and inflammatory M1 type macrophages as well as TAMs. In peritoneal macrophages, NRP2 is involved in phagocytosis. In microglia, NRP2 is polysialylated and remains confined in the Golgi compartment. Following LPS challenge, it rapidly translocates to the cell surface and is shed from the cells. The role of NRP2 in TAMs remains unknown.

Neuropilin-1 is also detected in alveolar, bronchial as well as intravascular macrophages. Recently, macrophages have been causally associated with and have emerged as therapeutic targets in several disease states. Macrophages are highly plastic and can switch their functional phenotype, depending on the cues they receive from the microenvironment. Broadly, they can be categorized into classically activated (M1) or suppressive (M2) types. These exhibit distinct gene signatures and cytokine profiles (128). While the M1 type macrophages are proinflammatory in nature, the M2 cells are proangiogenic and immunosuppressive and crucial for tissue remodeling and wound healing processes. Hence, any shift in this balance results in aberrant activity and a wide array of pathological conditions. However, classification of macrophages based simply on their gene signature and cytokine profile is overinterpreted because the two subtypes often co-exist and have overlapping gene profile(s). Recently, there has been a focus on the role of macrophages infiltrating solid tumors. Macrophages, which invade the tumor tissues, are polarized to an immune suppressive and protumorigenic type. These tumor-associated macrophages (TAMs) contribute to disease progression by releasing angiogenic factors and support the induction of immune tolerance (129–132). The recruitment of tumor-infiltrating macrophages to the avascular hypoxic core of a tumor is essential for their protumorigenic activities. NRP1 is expressed on TAMs and is crucial for their migration to the hypoxic niche of the tumor in response to Sema3A. Hypoxia induces the expression of Sema3A, which then interacts with NRP1 and PlexinA1/PlexinA4 on macrophages and triggers VEGFR1 activation and migration of macrophages to become TAMs. Once in the hypoxic environment, NRP1 is transcriptionally repressed in TAMs, which then lose their migratory capacity in response to Sema3A. The latter now elicits a “stop” signal through PlexinA1/PlexinA4 and entraps the TAMs in the hypoxic microenvironment of the tumor. Depletion of NRP1 arrested TAMs in the peripheral normoxic areas of tumors and abrogates their protumoral functions. In addition, antitumor T_H_1/cytotoxic T lymphocyte (CTL) response can be induced which reduced the tumor burden (133, 134) (Figure 3).

Interestingly, in neural progenitor cells, the binding of Sema3A with NRP1 recruits membrane VEGFR1 and induces cell repulsion. Further, a prolonged interaction between Sema3A and NRP1 induces apoptosis in the cells. Also, VEGF competes with Sema3A to bind to NRP1 and could antagonize the effects of the Sema3A/NRP1 axis. But it is not known how the same Sema3A/NRP1 axis can govern different pathways and induce different effects in two cell types. Interestingly, in a separate study undertaken by Wallerius et al. an opposite role of Sema3A was reported in the TME. These researchers observed that tumor cell-derived Sema3A binds to NRP1 on macrophages and negatively regulates their proliferation, while favoring M1 macrophage proliferation (135). Therefore, it is understandable why several malignancies downregulate Sema3A with advancement of the disease. Treatment with Sema3A switched tumor-associated immunosuppression toward immune activation, as documented from higher number of intratumoral inflammatory M1 type macrophages and increased proliferation of activated CD8^+^, NKT lymphocytes and significantly reduced tumor burden. NRP1 expression is also detected on alveolar macrophages adjacent to the cancer margin in patients with lung cancer (136). NRP1 expression is also found on peripheral blood mononuclear cells *in vitro* cultured in presence of colorectal cancer tissue (137). By contrast, Carrer et al. identified a novel subset of bone marrow-derived monocytes, which were CD11b^+^NRP1^+^Gr1^−^. When injected into tumors, these NRP1-expressing monocytes promoted tumor vasculature normalization. This resulted in less tumor vessel leakiness, better perfusion and decreased hypoxia and a significant reduction in tumor burden although no effect was observed on the proliferation of the tumor cells (138). Similarly, gliomas, a form of tumor arising in the brain or spinal cord have a high incidence (~30%) of infiltrating macrophages (GAMs). GAMs can be reprogrammed by tumor-secreted factors and promote tumor cell survival and proliferation. NRP1, which is expressed in GAMs has been associated with tumor promotion. Indeed, mice with GAM specific deletion of NRP1 resulted in slower tumor growth, reduced tumor vascularity and increased survival. Also, NRP1 depletion on GAMs repolarized them to a more antitumorigenic phenotype, characterized by an inflammatory cytokine gene expression profile. This conclusion is based on the treatment of mice bearing orthotopic glioma tumors with EG00229, a selective inhibitor of NRP1’s b1 domain (139). This indicates a probable role of NRP1 in macrophage immunosuppression. In agreement with this conclusion, myeloid cell-specific depletion of NRP1 increased sepsis in mice, a complex clinical disorder arising due to uncontrolled inflammatory response to microbial infection. This was attributed to LPS-mediated downregulation of NRP1 *via* the TLR4-NFκβ p50–65 pathways and exaggerated production of inflammatory cytokines in absence of NRP1 (140).

Multiple sclerosis (MS) is an autoimmune disorder characterized by progressive damage of the CNS. The murine experimental autoimmune encephalomyelitis (EAE) model is widely used to understand MS pathophysiology. It is well established that microglia polarization contributes significantly to MS progression and severity (141–145). Like macrophages, microglia cells can exist as a proinflammatory M1 or immunosuppressant M2 type. M1 polarized microglia has been causally associated with disease severity whereas M2 polarized microglia are associated with autoimmune disease recovery. Tuftsin, a peptide that arises due to cleavage of the Fc domain of the IgG heavy chain, can promote M2 polarization of microglia and alternative activation of T lymphocytes (146). Tuftsin binds to microglia NRP1 and triggers the canonical TGF-β pathway to promote M2 phenotype (147). In agreement with this, Tuftsin administration was rendered ineffective in EAE bearing mice when NRP1 was selectively ablated from microglia, resulting in persistent demyelination. In addition, the authors show that NRP1 in microglia engages in homophilic interaction with NRP1 on T_reg_s to trigger TGF-β release for immunosuppression. Another key function of microglia is to phagocytose cellular debris and facilitate recovery. Tuftsin can also increase microglia phagocytosis in a NRP1 dependent manner that can be abrogated by NRP1 depletion. However, in M1 microglia, the phagocytic capacity is not affected by NRP1 depletion in presence or absence of Tuftsin (148). It is interesting to note that Tuftsin shares sequence homology with exon 8 of VEGF. Taken together, this suggests that NRP1 has a role in the regulation of immune suppression and phagocytosis by microglia (Figure 3). Interestingly, Tuftsin administration *in vivo* can increase GAM infiltration of gliomas and increase tumor burden. However, it is not known if this effect is due to a Tuftsin-NRP1-mediated pathway. Interestingly, NRP1 in bone marrow adipocytes and macrophages engage in homophilic interactions to prevent production of granulocyte colony-stimulating factor and blocks the generation of mature granulocytes (149). One of the regulatory factors involved in aging of immunity is changes in the DNA methylation pattern over an individual’s lifespan that can result in age related impaired immunity. In monocytes isolated from elderly individuals, one of the most hypomethylated CpG sites is mapped to intron 2 in the *NRP1* gene (150). Further studies are needed to understand how this might affect the immunity in aged individuals.

The function of NRP2 in monocytic cells is still enigmatic. Previous studies, as well as, unpublished work from our lab reveals that NRP2, although not detected on monocytes, is strongly upregulated as the cells differentiate toward either M1 or M2 type macrophages *ex vivo*, in both humans and mice. NRP2 is also expressed on alveolar, bronchial, peritoneal, and intravascular macrophages in mice (136). NRP2 positive macrophages have been detected in patients with lung cancer as well in mouse mammary tumors (136, 151). Previous work from our lab has documented a role for NRP2 in maintaining high endocytic activity on cancer cells by affecting the maturation of early to late endosomes, thereby favoring oncogenic activity of the tumor cells (152). The processes of endocytosis and phagocytosis are similar, both recruiting similar molecules during the maturation stages. Indeed, ongoing work in our lab suggests a requirement for NRP2 in regulating phagocytosis by macrophages. Stamatos et al. reported a progressive loss of polysialylation in monocytes and monocyte-derived cells as they migrate to pulmonary and peritoneal sites of inflammation (153). Polysia has an important role in migration and cell–cell communication between immune cells and hence immunity. We discussed the role of polysialylated NRP2 in DCs in earlier sections. Interestingly, peritoneal macrophages do not express polysia, but it is re-expressed on NRP2 when cells are cultured. Removal of polysia enhances macrophage phagocytosis of *Klebsiella pneumoniae* by (Figure 3). However, it is not clear if this effect could be attributed to NRP2 (153). It will be interesting to see if re-expression of polysia has a role in interaction of peritoneal macrophages with T lymphocytes following phagocytosis or for eliciting immune response. Polysialylated NRP2 has also been reported in microglia. Unlike DCs where polysialylated NRP2 is expressed on the membrane, in a recent article, Werneburg et al. identified polysialylated NRP2 to be confined in the Golgi compartment of microglia, which is quickly mobilized to the cell membrane during initial phase of LPS stimulation and is eventually lost. Upregulation of inducible nitric oxide synthase and increased production of NO is a hallmark of LPS challenged microglia. Exogenous addition of polysia blunted the production of NO in microglia in response to LPS, indicating a probable role of polysia on NRP2 in negative feedback regulation of proinflammatory responses (154). Further, NRP2 polysia is detected in the cell culture supernatant following LPS addition to culture indicating metalloproteinases-mediated cleavage of the protein and shedding. Interestingly, IL-4, which polarizes microglia toward an anti-inflammatory phenotype, does not affect the polysialylated pool of NRP2 in the Golgi compartment (155) (Figure 3). However, one must note since addition of free, soluble polysia could abrogate LPS induced NO release, this indicates this process does not depend on the polysia carrier. However, why polysia is maintained on multiple proteins and how it modulates an inflammatory response is still unclear. Also, given the brief duration of the membrane presentation of polysia-NRP2 during early LPS challenge raises the question if it regulates interactions with other proteins or has other functions questions that need to be investigated in greater depth in the future.

In summary, a critical role for NRP1 in TAMs has been documented and may emerge as a novel therapeutic target. However, the expression pattern and function of NRP2 in TAMs is not clear. Although NRP2 is expressed on TAMs (136, 151), no clinical-pathological data is available to indicate a correlation between NRP2 expression on TAMs and disease prognosis. Such studies would help design novel therapeutic strategies for immune targeting NRP2 to TAMs. One confounding factor in studying the role of NRP2 in macrophage is the two NRP2 splice variants. It is not known if the two isoforms, NRP2a and NRP2b have redundant or distinct and opposite functions in macrophages, in different cellular contexts. Hence, additional studies are needed to understand the role and function of the splice variants in TAMs and successfully targeting them for treatment of pathological conditions.

## NRPs in Osteoimmunology

Bone is a dynamic tissue that is continuously remodeled by osteoclasts and osteoblasts. Monocytes can differentiate into bone resorbing osteoclasts; whereas, the bone forming osteoblasts are differentiated from mesenchymal stem cells. Bone remodeling is a complex autocrine and paracrine interaction between osteoclasts and osteoblasts that contribute to the maintenance of bone tissue. Any shift in this tight coupling will result in various pathological conditions, such as osteoporosis. Further, many cancer related deaths result from bone metastasis. Detached tumor cells from primary tumor engage in a complex cross talk with both osteoclasts and osteoblasts that can provide a premetastatic niche supporting the arrest and growth of disseminated cancer cells. Hence, it is important to understand the interaction of tumor cells with bone micro niches and the molecular regulators involved. Recent reports indicate that the expression of Semaphorins and their receptors (NRPs, Plexins) have important role in bone remodeling. For instance, NRP1 provides osteoprotection by binding Sema3A, impairing RANKL-mediated osteoclast generation by negatively regulating ITAM and RhoA pathways, while promoting the formation of osteoblasts through the canonical Wnt-signaling pathway (156). Sema3A is produced by osteoblasts and can inhibit osteoclastogenesis from osteoclast precursor cells (156, 157). It also promotes osteoblast formation from bone marrow mesenchymal stem cells, thereby having a dual role in bone homeostasis (158). A recent report by Zhang et al. provided evidence that the discoidin domain receptor 2 promotes binding of NRP1 with PlexiaA1 and inhibits osteoclastogenesis (159). NRP1 has also been implicated in peri-prosthetic osteolysis (PPO), which occurs in response to prosthetics and eventually leads to bone loss and replacement failure. In human PPO samples, NRP1 was detected in multinucleated cells containing prosthetic wear particles (160). Further studies were needed to decipher the role of NRP1 in PPO. NRP2 is also expressed on both bone marrow-derived osteoclast and osteoblast compartments *in vitro* as well as *in vivo*. A deficiency in NRP2 expression can severely reduce trabecular bone mass. This is accompanied by an increase in osteoclastic number and reduced osteoblast formation (48). Further, NRP2 expression is elevated in human osteosarcoma patients (the RNA level) and correlated with hypervascularity, one of the key features of osteosarcoma and a poor prognosis (161). In a recent study, a tissue microarray analysis with 66 osteosarcoma patients identified NRP2 as a predictive marker for poor overall, metastasis free and progression free survival but did not detect any predictive value for NRP1(162). NRP2 was also overexpressed on osteosarcoma cell lines and depletion of NRP2 through downregulation of active Wnt-signaling pathway significantly reduced tumor burden and metastasis by osteosarcoma cell lines (163). Together, these data suggest that both NRP1 and NRP2 are crucial for osteoclastic and osteoblastic activity. Many cancers including prostate, breast, lung, kidney, stomach, bladder, uterus, thyroid, and colorectal metastasize to the bone. Bone metastases usually signify an advanced and incurable disease. The complex cross talk between disseminated tumor cells from the primary lesions with the osteoclasts and osteoblasts result in either osteolytic or osteoblastic lesions. This results in the release of growth factors from the bones and eventually creates the metastatic niche for tumor cells. It will be interesting to test the function of NRP1 or NRP2 in regulating bone metastasis and whether this can emerge as a therapeutic target in the future.

## Multifaceted Role of NRPs in T Cells

T cell-mediated adaptive immune response is a complex and tightly coordinated process and is indispensable for controlling foreign pathogens and malignant tumor cells. Thymocyte (T cell) development and maturation is a tightly regulated process. T cell subsets exhibit unique gene signature profiles regulating T-cell differentiation and maturation based on the growth factor and cytokine milieu. Aberrant development, activation, and dysfunction of T cells are detrimental to host response to foreign invaders and can result in autoimmunity, cancer and other pathologies. Numerous reviews have highlighted multiple aspects of T cell biology and how they can be harnessed for effective immunotherapy of chronic, infectious, and neoplastic diseases (164–178). Here, we discuss the expression of NRP1 and NRP2 and how they regulate the development, maturation, and effector functions of different T cell subsets.

## Expression and Function of NRP1 in Different T Cell Subsets

Although extensively studied over the past few years, until now the role of NRP1 in T cell biology remains unclear. However, its expression has been frequently associated with immune suppression. In murine thymus, NRP1 expression is detected as early as day 12.5 of gestation. In adult mice, its expression is detected at multiple stages of thymocyte maturation. For instance, it is detected in double positive (DP), double negative (DN), and the regulatory T cell (T_reg_) compartments but rarely detected in the CD4^+^ or CD8^+^ single-positive (SP) populations. However, deletion of NRP1 did not affect normal thymocyte populations (53). During T cell development, bone marrow-derived progenitors migrate to the thymus, where they travel through the cortex and medulla in response to an array of chemoattractants as well as chemorepulsants. During this time, thymocytes interact with thymic epithelial cells (TECs) and DCs, a crucial step for positive and negative selections, which eventually results in naïve CD4^+^ or CD8^+^ T cells. Immunohistochemical analysis has revealed a strong expression of NRP1 on human TEC. Interestingly, only a minority of the intrathymic T cell population is NRP1 positive. Cell–cell adhesion between TEC and thymocytes further increases NRP1 expression on thymocytes that could be attributed to IL-7 secreted from TEC and TCR engagement, both of which can increase NRP1 expression on thymocytes. This suggests that NRP1 is important for adhesion between TEC and intrathymic thymocytes and this can be abrogated by Sema3A. The latter is present in thymocytes and TECs and is upregulated on thymocytes following TCR engagement. In axonal guidance, the NRP1/Sema3A axis regulates actin cytoskeleton reorganization. Hence, it is possible that the NRP1/Sema3A axis in thymocytes modulates similar actin cytoskeleton reorganization and induces their loss of adhesion to TECs. Further, the Sema3A/NRP1 axis negatively regulates thymocyte migration, thereby acting as a chemorepulsant. Overall, the study put forth a hypothetical model, IL-7 secreted from TEC upregulates NRP1 in thymocytes supporting adhesion to TECs. Following this interaction, TCR engagement increases NRP1 and Sema3A expression, which then detach the thymocytes from TECs and modulate their migration (51, 179).

## Regulatory T Cells

**Figure 4:**
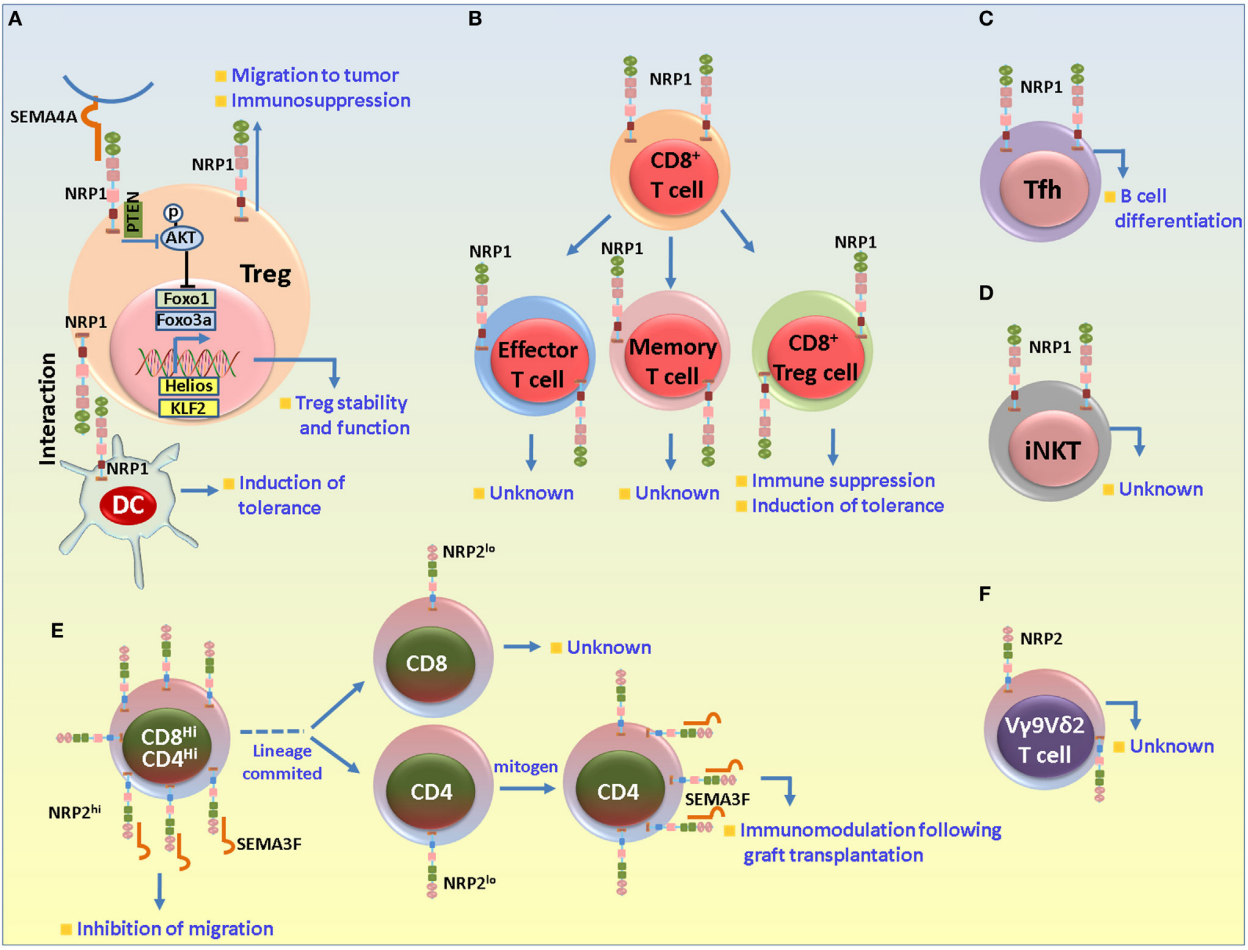
Role of neuropilin-1 (NRP1) and neuropilin-2 (NRP2) in different T cell subsets. **(A)** NRP1 in regulatory T cells (T_reg_s). NRP1 has been mainly associated with suppressive functions in T_reg_s. NRP1 is important for the formation of immunological synapse between dendritic cell (DC) and T cells. In absence of danger signal, T_reg_s preferentially interact with DCs to establish immune tolerance. NRP1 regulates the stability and functional stability of T_reg_s. Sema4A secreted from DCs binds to NRP1 and recruits PTEN to restrain Akt phosphorylation to facilitate the nuclear translocation of Foxo3a and favor T_reg_ survival, stability and quiescence. NRP1 also plays a role in the migration of T_reg_s to the tumor microenvironment in response to tumor cell-derived vascular endothelial growth factor. **(B)** NRP1 in CD8^+^ T cells. NRP1 is detected in the effector and memory CD8^+^ cells (following exposure to self-antigens under non-immunogenic conditions) and may be involved in maintenance of immune homeostasis in absence of any danger signal. NRP1^+^CD8^+^ T_reg_s are also detected in neoplasms and may be important for imparting immunosuppression and disease progression by impairing CD8^+^-derived functions. **(C)** NRP1 in T follicular helper (Tfh) cells. NRP1 is expressed in a subset of Tfh cells and is important for B cell differentiation. **(D)** NRP1 in invariant natural killer T (iNKT) cells. NRP1 is detected in recent thymic emigrant iNKT cells, although its exact role in these cells is not clearly understood. **(E,F)** NRP2 in T cell subsets. NRP2 is highly expressed by the CD4^+^CD8^+^ double positive immature thymocytes, but its expression is reduced to baseline in the CD4^+^ or CD8^+^ single-positive cells. In the immature cells, NRP2 in concert with Sema3F and PlexinA1 negatively regulates the migration of the immature thymocytes in response to CXCL12 and S1P1. Following lineage commitment, NRP2 expression decreases to facilitate the egress process from the thymus. Its expression is once again induced in the mature cells following exposure to mitogen and is involved in immunomodulatory functions during graft transplantation. NRP2 is also detected in the VY9Vδ2 cells in ALL and non-Hodgkin’s lymphoma in the resistant tumor samples.

Regulatory T cells are potent immunosuppressive cells that are associated with immune homeostasis, tolerance to self-antigens and the prevention of autoimmune disorders as well as pathological conditions including cancer, allergy, graft tolerance *etc*. This is attributed to their secretion of anti-inflammatory cytokines such as IL-10, TGF-β, granzyme A/B, expression of checkpoint inhibitors, and the impairment of effector T cell function through metabolic disruption and DC immunomodulation (180). They arise in the thymus by positive selection [natural T_reg_ (nT_reg_)] or are induced peripherally from CD4^+^ T cells [peripherally induced T_reg_ (iT_reg_)]. The regulatory role of T_reg_ has been well documented in several inflammatory diseases in mice and humans and is usually identified based on the co-expression of CD4, CD25, and forkhead box P3 (Foxp3). NRP1 is constitutively expressed on the membranes of murine CD4^+^CD25^+^Foxp3^+^ T_reg_ cells, irrespective of their activation state. Its expression correlates with their suppressor phenotype and hence is considered to be a murine T_reg_ marker (52, 53). We previously discussed a role for NRP1 in the initiation of ISs between DC and T lymphocytes. This interaction is a key determining factor for IS formation. While brief interactions are common while scanning for antigens, long interactions eventually result in IS formation. Sarris et al. reported that iDCs preferentially formed immune synapse with T_reg_s, which is abrogated when anti-NRP1 is added. The ectopic expression of NRP1 on T Helper (T_H_) cells increased their sensitivity to cognate antigen that is mediated by homophilic interactions between NRP1 expression on iDC and T_reg_s. These findings suggested that under normal physiological conditions when antigen is limited, or, a danger signal is absent, NRP1 provides a heads start signal by preferentially interacting with T_reg_s and preventing T_H_ cellular interactions with DCs, helping establish tolerance (181) (Figure 4).

Several reports have established the role of TGF-β and IL-2 in T_reg_ cell development and function. For instance, TGF-β can induce the formation of T_reg_ from CD4^+^ T cells as well as their development in the thymus. IL-2 also has a major role in regulating the stability of TGF-β induced iT_reg_s *in vivo* (182–185). In a recent study, a synergistic effect of blocking TGF- β and IL-2 in T_reg_s development using dnTGF-βRII IL2ra^−/−^ mice was shown where TGF-β and IL-2 signaling were blocked in T cells. These mice spontaneously developed autoimmune diseases at 3–4 weeks of age. Interestingly, the iT_reg_s from these mice exhibited an activated T_H_1-like phenotype while the thymus-derived nT_reg_s had reduced suppressive functions. However, these T_reg_s did not express NRP1 and had a defective follicular T_reg_s development, resulting in increased number of follicular T helper (Tfh) cells, enhanced germinal center responses and concomitant plasma cell infiltration. These data indicate that NRP1 expression on T_reg_s depends on TGF-β and IL-2 signaling and that they may have additive activity (186). In addition, NRP1 on T_reg_s can bind the latent form of TGF-β from the surrounding tissue fluid or plasma and impart further immunosuppression (187).

Several studies have related NRP1 expression on T-cells with immunosuppression. CD4^+^ T cells from mice with an NRP1 knockin and a disrupted binding site for Semaphorins, were hyperproliferative following exposure to CD3 as a mitogen and to allogenic DCs. NRP1 was also selectively induced on activated CD4^+^ T cells in skin draining pLNs, which then facilitated their migration to cutaneous sites of inflammation. In EAE characterized by infiltration of CD4^+^ T cells and an aberrant inflammatory response against myelin components, deletion of NRP1 on CD4^+^ T cells had an increase in the inflammatory T_H_17 lineage cells and diminished T_reg_ cells, resulting in EAE severity (188). The induction of tolerance is the Holy Grail in transplantation biology and is crucial for graft survival. Hyperactivated T cell response is a major limiting factor in graft acceptance. During graft rejection, there is a decrease in NRP1 expression in the T_reg_ population; adoptive transfer of CD4^+^NRP1^+^ T cells to mice receiving MHC mismatched heart and skin allograft resulted in prolonged graft survival (189, 190). NRP1 expression in CD4^+^CD25^−^Foxp3^+^ T_reg_s isolated from the decidua of pregnant women is crucial in inducing immune tolerance against semi-allogenic fetus (191). However, in contrast to the above mentioned beneficial role of NRP1 in graft acceptance, in their recent published abstract at the American Transplant Congress 2016, Lee et al. argued that NRP1 is upregulated on effector T cell compartment following allogeneic activation and contributes to graft rejection in a major and minor mismatch setting, as evident from the upregulation of NRP1 expression on effector CD4^+^ T helper and CD8^+^ T cell subsets. Indeed, depletion of NRP1 on CD4^+^ T cell lineage cells significantly prolonged graft survival in a minor mismatch setting. Interestingly, in mice that either did not or received syngenic grafts, NRP1 was mainly detected in T_reg_s, and much less on the conventional CD4^+^ and CD8^+^ subsets. In summary, although NRP1 expression on T-cells promotes immunosuppression, its role may vary with different T cell subsets isolated from different organs and under different experimental settings.

The phenotypic characterization of naïve and activated T_reg_s is complex. Various markers have been proposed to identify naïve or activated T_reg_s in different tissues and extensively reviewed elsewhere. However, the most frequently used markers for T_reg_s include CD45RO, Foxp3, CD4 and CD25. One disadvantage is that these markers fail to distinguish between the thymic-derived nT_reg_ and peripherally induced iT_reg_s. Since these T_reg_s have non-overlapping functions, it is essential to identify proper markers, which can distinguish between these two subpopulations and be used for designing selective therapeutic targeting strategies. Recently, Helios, a transcription factor and member of the Ikaros family was reported to be specific for nT_reg_s and to distinguish between these two subsets (54, 192). However, Helios deficiency has no effect on the development, survival and functional phenotype of nT_reg_s, resulting in a question of its usefulness as a T_reg_ marker (193, 194). Several papers reported high NRP1 expression on nT_reg_s compared with iT_reg_s (195, 196) and a correlation of NRP1 expression with Helios on Foxp3^+^ T_reg_ population(s). In agreement with this, NRP1 expression was not detected on iT_reg_s isolated from the large and small intestine of mice (196), where components of the microbiota have a major role in the generation of iT_reg_s. These two reports suggest that NRP1 expression can distinguish between nT_reg_s and iT_reg_s. However, NRP1 may not be an optimal marker under all conditions. iT_reg_s isolated from the spinal cord of mice afflicted with either EAE or chronic lung inflammation have a high expression level of NRP1; whereas iT_reg_s in the secondary lymphoid tissues from the same animals have low expression levels of NRP1 (196). In brief, iT_reg_s that form in a chronic inflammatory environment have upregulated NRP1 expression in contrast to iT_reg_s generated under tolerogenic conditions. One possible explanation is that the inflammatory cytokines in the inflamed tissues favor the expression of NRP1 in iT_reg_s. However, the exact function of NRP1 in these T_reg_s is unknown. Mice lacking NRP1 expression on thymocytes do not have a defect in T_reg_ generation or development of any autoimmune disorders. Further, Helios may be a better marker over NRP1 to identify nT_reg_s and that they expressed higher level of some of the genes associated with T_reg_s (e.g., IL-10, CTLA-4) and were more stable from apoptosis in thymus (54). In this study, the authors observed a higher frequency of Helios^+^ cells over NRP1^+^ cells among the CD4^+^CD25^+^ and CD4^+^CD25^+^Foxp3^+^ populations in the thymus. Similar arguments have been made in a study where NRP1 was insufficient to unambiguously distinguish between an intrathymic or extrathymic origin of T_reg_s. Here, several genetically engineered mouse models with a compromised ability to generate nT_reg_s or iT_reg_s were used revealing that high or low level of NRP1 and/or Helios may not be sufficient to identify the origin of T_reg_s (55). Moreover, the peripheral TCR repertoires of CD4^+^Foxp3^+^NRP1^high^ (or Helios^high^) and CD4^+^Foxp3^+^NRP1^low^ (or Helios^low^) cells were similar to each other. Overall, studies to date suggest that NRP1 expression on T cells may promote immune suppression, although its role as a marker to distinguish different subsets of T_reg_s is controversial.

One important caveat to these studies is that NRP1 expression on human T_reg_s differs significantly from murine T_reg_s. Although NRP1 is a murine T_reg_ marker, several studies have argued against the use of NRP1 to identify human T_reg_s. Milpied et al. observed a low expression of NRP1 on both CD4^+^ T and CD4^+^CD25^+^Foxp3^+^ T_reg_s isolated from the peripheral blood, thymus, spleen, pLN, and tonsil of human donors. Although T_reg_s in the thymus expressed more NRP1 than CD4^+^ cells, this pattern of expression was reversed in cells isolated from tonsil; however, none of these observations achieved statistical significance. Also, NRP1^+^ T cells identified in the secondary lymphoid organs comprised only a very minor population of the entire T cell pool and mostly associated with non-regulatory phenotype (197). However, several groups have reported NRP1-expressing T_reg_s in humans under different pathological conditions, including cancer, as discussed later in this section. NRP1^+^ T_reg_s were also detected in the synovium of patients suffering from rheumatoid arthritis, osteoarthritis, and bronchoalveolar lavage of patients suffering from chronic obstructive pulmonary disease (198, 199). NRP1-expressing T_reg_s were scarce in kidney graft biopsies from patients who showed acute rejection and abundant in those who did not reject the graft (200). This shows NRP1, similar to murine T_reg_s, exerts a similar immunosuppressive role in human T_reg_s.

The exact function of T_reg_s in cancer is a contentious subject. They have been detected in circulation as well as TMEs in various tumors, in human as well as mice. Over the years, studies from different groups have highlighted on the pivotal role played by T_reg_s in dampening antitumor immune response through their interaction with the other immune cell infiltrates in the TME. Therefore, they pose a substantial threat to antitumor therapies and correlate with poor clinical outcome (201–208). NRP1-expressing lymphocytes have been detected in several malignancies and associated with a protumorigenic role (Figure 4). For instance, a high level of NRP1 expression was detected on CD3^+^CD4^+^ lymphocytes isolated from colorectal cancer liver metastasis samples. NRP1 was expressed in Helios^+^ as well as Helios^−^Foxp3^+^ T cells and its expression correlated with that of CD25, indicating these NRP1-expressing cells were Tregs. Further, the group also detected NRP1^+^ T_reg_s in the peripheral blood isolated from patients suffering from pancreatic ductal adenocarcinoma (PDAC) and colorectal cancer liver metastasis. Interestingly, NRP1^+^ T_reg_s were not detected in the peripheral blood of a patient with metastatic PDAC who underwent resection of the tumor (3, 137). Direct evidence of functions of NRP1 in T_reg_s was documented by Hansen et al.; the authors showed that VEGF secreted by tumor cells bind to NRP1 on T_reg_s and mediates their migration to the TME. Depletion of NRP1 in CD4^+^ T cells resulted in breakdown of tumor induced tolerance and activated antitumor immune response in tumor bearing mice, evident from increased CD8^+^ and reduced T_reg_ cell infiltration into the TME, lesser tumor burden and improved tumor free survival. Adoptive transfer of NRP1^+^ T_reg_s abrogated this effect (209). Immunophenotypic and functional assessment of the immune state in women diagnosed with metastatic cervical cancer versus those still in the early stage of the disease revealed an enrichment of NRP1^+^ T_reg_s in the tumor-draining lymph nodes (TDLNs). These cells were potent in suppressing T cell response. Immunohistochemical analysis also showed an increased secretion of VEGF, a natural ligand for NRP1 expressed on the T_reg_s, thereby facilitating the formation of an overall tolerogenic and immunosuppressed environment in the lymph nodes. This in turn favored tumor metastasis (210). NRP1 is also upregulated on T_reg_s isolated form the peripheral blood of patients with CLL (106). That NRP1 on T_reg_s plays immunosuppressive role in tumor advancement was documented by a decrease in the number of NRP1^+^ T_reg_s in the TDLNs of cervical cancer patients who received preoperative chemotherapy and resulted in favorable outcome, indicating a switch to antitumor immune response activation. In addition, NRP1^+^ T_reg_s also decreased in patients with advanced cervical cancer receiving a combination of low dose radiation and chemotherapy, in comparison to those who only received chemotherapy (211, 212). Also, there was a significant reduction in the NRP1^+^ T_reg_s in the peripheral blood of CLL patients who underwent treatment with the drug thalidomide. This drug tends to reduce the level of VEGF in patients. Hence, NRP1 downregulation in T_reg_s following treatment with thalidomide may arise as a direct or indirect effect of the drug (106). This indicates a tumor promoting role for NRP1 in T_reg_s and poor clinical outcome. Interestingly, there was a further increase in the number of NRP1^+^ T_reg_s in the TDLN of patients receiving chemotherapy in combination with high dose radiation. This indicated even a change in radiation dose in neoadjuvant therapy regimens can yield opposite results and lead to further immunosuppression and disease progression (211). Large granular lymphocytic leukemia (LGL) is a disorder characterized by the malignant expansion of either CD3^+^CD8^+^ or NK cells. It gives rise to autoimmunity and autoimmune cytopenias. A novel somatic NRP1 mutation (V391M) was reported in a fraction of patients diagnosed with LGL, indicating a potential role in disease outcome (213). Recently it was reported that intratumoral T_reg_s are a mixture of nT_reg_s and iT_reg_s. However, it is not clear how the two subpopulations may differentially regulate tumor growth. Treatment of tumors with IL-12 adjuvant therapy results in activation of helper T and cytotoxic T cells and regression of T_reg_s. However, within a few days, there occurs a massive reinfiltration of T_reg_s into the TME, and the immunosuppressive phenotype is quickly restored (214–216). This spike in T_reg_ reinfiltration is driven by CD4^+^Foxp3^+^NRP1^low^ iT_reg_s, which is then followed by NRP1^hi^ nT_reg_s (217). NRP1 also regulates the stability and function of T_reg_s in the TME through modulation of Akt activation. We have discussed earlier that pDCs comprise an important immune infiltrate in various TMEs. They are a rich source of Sema4A. The latter binds NRP1 on T_reg_s *via* the PDZ binding motif on NRP1 and recruit phosphatase and tensin homolog (PTEN) at the IS to restrain Akt phosphorylation. Phosphorylation of Akt prevents the nuclear trafficking of Foxo transcription factors, which are important for T_reg_ cell stability and function. Therefore, NRP1/Sema4A axis prevents Akt phosphorylation and promotes nuclear translocation of Foxo3a and contributes to T_reg_s survival, stability, quiescence and suppresses several lineage commitment markers (218) (Figure 4). Further, depletion of NRP1 in intratumoral T_reg_s renders them functionally fragile characterized by increased IFN-γ production and T_H_1 markers. These fragile T_reg_s can then induce fragility in the surrounding T_reg_s in a feed forward loop. The net result is activation of antitumor immune response, increased intratumoral CD8^+^ T cell infiltration and reduced tumor burden (219). In a recent report, the authors attributed T_reg_-derived NRP1 function to be largely under the control of IL-10, a known immunosuppressant and anti-inflammatory cytokine. IL-10 abrogation significantly dampened T_reg_-derived NRP1 signaling, concomitantly facilitating T_H_1 and T_H_17 type activation. This resulted in increased production of IFN-γ, IL-17, and CD8a and reduced tumor burden (220). Taken together, these findings place NRP1 as a potential candidate for targeting T_reg_s in the treatment of diseases such as cancer.

## Follicular Helper CD4^+^ T Cells

T follicular helper cells are a specific subset of T cells and are important for the development and survival of antigen specific B cells in the secondary lymphoid organs. They are mainly found within the B cell follicles of secondary lymphoid organs where B cells proliferate to give rise to plasma and memory B cells. Antigen specific interaction between MHC-II-peptide loaded cognate B and TCR on Tfh cells results in bidirectional signaling, which eventually facilitates B cell differentiation (221–224). NRP1 is expressed by a subpopulation of Tfh cells in the secondary lymphoid organs in humans and its expression can be induced *in vitro* by interaction with autologous memory B cells and correlated with the plasma B cell precursors (plasmablasts). This indicated a role for NRP1 on Tfh cells in B cell differentiation. Also, NRP1^−^ and NRP1^+^ Tfh cells *ex vivo* similarly expressed most of Tfh associated genes, yet showed differential expression of some of the cytokines (for example, less IL-10 and more IL-4) and surface receptor genes. NRP1 was also highly expressed by Tfh cells following contact with cognate B cells in one out of five patients suffering from angioimmunoblastic T cell lymphoma (AITL) and correlated with the terminal differentiation of B cells (56). However, a bigger panel of patients needs to be tested to confirm and understand how NRP1 contributes to disease pathology in this clinical setting. Since NRP1 expression was not detected in the B cells even after coculture with Tfh cells, it seems not to function as a homotypic adhesion molecule during the formation of synapse between B cells and T cells. It is, however, possible that it is required for migration of Tfh cells to and their retention in the germinal center of secondary lymphoid organs. Based on these findings, it can be speculated that NRP1 may be used as a Tfh marker and prognostic factor for disorders associated with aberrant Tfh functions.

## CD8^+^ T Cells

CD8^+^ T cells are a subset of T cell family known for their cytotoxic properties. They detect infected and transformed cells and mount robust immune response for elimination of those cells. However, for maintenance of normal homeostasis, establishment of peripheral CD8^+^ T cell tolerance is crucial to prevent excessive activation of the former and limit their cytotoxicity to self-cells. Over the last few years, researchers have once again started to focus on this subpopulation in various pathologies. Various aspects of CD8^+^ lymphocytes have been covered in several reviews (165, 225–230). Briefly, CD8^+^ T cell response progresses through three different phases: clonal expansion of antigen specific CD8^+^ T cells into effector cells, contraction and death and finally, formation of long term memory cell population. Following viral infection, NRP1 was moderately upregulated in effector and memory CD8^+^ T cells compared with naïve cells (231). Recently, NRP1 expression has also been reported in CD8^+^Foxp3^+^ cells in the intestinal mucosa following exposure to gut specific antigen (232). These cells could suppress CD4^+^ T cell proliferation *in vitro*. Liver sinusoidal endothelial cells, a population of non-professional APCs in the liver, cross present exogenous antigens circulating under non-inflammatory conditions to generate a unique population of NRP1^+^CD8^+^ memory T cells. These cells evade clonal deletion and attain a memory cell like phenotype and home to lymphoid tissues. These cells are different from conventional non-responsive or exhausted T cells but differentiate to effector CTLs following re-encounter with antigen presented by immunogenic DCs, even after prolonged period of absence of antigen. This type of T cell priming by non-professional APC will enable clearance of pathogens that may colonize the body by escaping initial innate immunity and induction of peripheral tolerance during the initial phase of infection. However, the function of NRP1 in this T cell subset is still unknown (233). Interestingly, in a separate study, Jackson et al. demonstrated that CD8^+^ T cells upregulated NRP1 expression following exposure to self-antigens in the liver. However, NRP1 neither facilitated nor hindered the tolerant phenotype and peripheral deletion of these cells. In addition, adoptive transfer of NRP1 ablated T cells did not provide any survival benefit in tumor bearing mice, further indicating NRP1 was dispensable for tolerant phenotype in CD8^+^ T cells (234). It is now well accepted that CD8^+^ T cells are antitumorigenic and are important for tumor regression. However, in the TME, under chronic exposure to tumor antigens and other negative costimulatory molecules as well as negative immunomodulation by other infiltrating immune cells, they differentiate to a dysfunctional and corrupted memory cell population. Many cancer vaccine strategies fail because they fail to correct or reset these dysfunctional CD8^+^ memory T cells. A population of CD8^+^CD25^+^Foxp3^+^ T_reg_s was found to be augmented in the tissues of patients diagnosed with colorectal tumor. NRP1 was also detected on tumor-infiltrating CD4^+^ and CD8^+^ T cells isolated from patients undergoing resection for metastatic melanoma and correlated with antigen experienced CD45RO phenotype. These NRP1^+^CD8^+^ T_reg_s isolated from two representative patients, when stimulated with CD3/CD28, showed decreased proliferation *ex vivo* (234). NRP1 was also detected on exhausted CD8^+^ T cells in chronic infection. It remains to be seen if NRP1-expressing CD8^+^ T cells in cancer represent exhausted CD8^+^ cells. Hence, the exact role of NRP1 under these conditions still remains elusive and require extensive investigation.

## NKT Cells

Natural killer T cells are a specialized family of T cells that recognize and respond to lipid antigens and play an important role in immune responses. Studies have shown that these cells can have protective as well as pathogenic role in various diseases. There are excellent reviews, which have summarized the current knowledge about these cells (235–239). Briefly, based on their T cell receptor (TCR) repertoire, they are categorized into type I and type II NKT cells, the former being CD4^+^ or DN, and produce an array of proinflammatory as well as anti-inflammatory cytokines, which can induce either T_H_1 or T_H_2 type responses and thus affect all other immune cell types. Type II NKT cells, on the other hand, have more diverse TCR repertoire and are difficult to characterize. However, they can also produce a wide variety of effector molecules. Type I NKT cells, also known as invariant natural killer T (iNKT), have been reported to play an important role in cancer. Their number is usually decreased in solid tumors; increased infiltration of iNKT cells correlates with activation of antitumor immune response and hence better prognosis (240–245). NRP1-expressing iNKT cells have been detected in thymus and peripheral lymphoid organs. NRP1^+^ iNKT cells comprised the recent thymic emigrant population; however, NRP1 was not detected in long-lived, mature iNKT cells. The NRP1^+^ recent thymic emigrant iNKT cells were extremely potent in producing IL-17, but not IFN-γ or IL-4, a feature of the mature cells (57, 58). However, questions still remain under scrutiny as to why NRP1 is selectively expressed in the recent thymic emigrants only. Also, if NRP1 plays a role in NKT cell development and egress from the thymus still is unknown. NRP1 can bind and signal through TGF-β1, the latter being crucial for iNKT cell development (246). Hence, it is possible that NRP1 expression on iNKT cells increases their sensitivity and response to TGF-β1 and thus regulates their development. NRP1 can also participate in cell–cell communication when presented in trans. A recent study documented the importance of stable cognate interaction between iNKT cells and macrophages for eliciting response to lipid antigens in the lymph nodes (247). NRP1, which is expressed by lymph node resident iNKT cells and macrophages, may be involved in homophilic interaction between these two cell types and affect immune response. Further studies need to be undertaken to identify the ligands that bind with NRP1 in the recent thymic NKT cell emigrants and how it regulates or correlates with IL-17 production. NRP1 expression is also detected in a subset of IL-10-producing iNKT cells with suppressive phenotype (248). Hence, it is possible that NRP1 is required for regulating immune responses in these cells. In depth studies are needed to clearly understand the role and function of NRP1 in iNKT cells, the pathways it governs and how it can be targeted in diseases.

## Expression and Function of NRP2 in Different T Cell Subsets

As in case of other immune cell types, NRP2 is comparatively less studied in T cells. Constitutive expression of NRP2 in TECs and developing T cells in the human thymus has been reported. Interestingly, in contrast to NRP1, expression pattern of NRP2 varied in the CD4/CD8-defined subsets. NRP2 was highly expressed in the CD4^+^CD8^+^ DP T cells. Its expression decreased in SP CD4^−^CD8^+^ and CD4^+^CD8^−^ cells as they gradually became lineage committed. A similar pattern was observed for Sema3F expression, which is a ligand for NRP2. CXCL12 is an important chemokine in thymocyte development. NRP2/Sema3F axis was reported to impair thymocyte migration in response to CXCL12 by inhibiting cytoskeleton reorganization. Interestingly, Sema3F-mediated impairment of migration toward CXCL12 was also observed in the mature SP cells, where NRP2 expression was low. This suggested the involvement of receptor/s other than NRP2 here. NRP2/Sema3F axis also inhibits thymocyte migration in response to S1P1, a chemokine with well-documented role in thymocyte egress from the thymus. Since S1P1 is crucial for thymocyte emigration, it is likely that NRP2 downregulation in SP T cells occurs to facilitate the egress process (249). Studies have indicated that Sema3F in concert with NRP2 and PlexinA1 can cause depolymerization of F-actin filaments in cells through inactivation of RhoA GTPase, thereby causing cytoskeleton collapse and dampened migratory response (250). It may be possible that similar pathways contribute to the regulation of thymocyte migration. NRP2 expression has also been detected in mouse thymic compartments (251, 252). However, it is still not clear if NRP2 plays a similar role in murine thymocytes. NRP2 and Sema3F were also detected in T-cell acute lymphoblastic leukemia (T-ALL) and T-cell lymphoblastic lymphoma (T-LBL) samples and modulated migration in response to CXCL12 and S1P in a similar fashion in the former (249). T-ALL and T-LBL are manifested by malignant proliferation of thymocytes whose differentiation is arrested. This indicates a potential role of NRP2 in these two neoplasms. Hence, the findings may be relevant for designing drugs to treat disorders involving thymocytes.

γδ T cells are a unique subset of T lymphocytes family. They arise from the common thymocyte progenitors; however, their TCR complex comprises of γ and δ chains and do not usually express the lineage markers, CD4 or CD8. They recognize distinct antigens in MHC independent manner and act as a bridge between the innate and the humoral response in host. They can exist as different subpopulations, each with specific locations and repertoire of γδ chains. The subset, which express δ2 chain usually co-express γ9, hence called Vγ9Vδ2 cells. These comprise 50–95% of all γδ T cells in peripheral blood. They can act as professional APCs, recruit macrophages, trigger DC maturation, produce inflammatory cytokines, and exhibit cytotoxic properties. Given their non-MHC restricted cytotoxicity toward a wide variety of tumor cells, Vγ9Vδ2 T cells are considered an attractive target for immunotherapy, especially for malignancies of hematopoietic origin. Several clinical trials have been carried out, either by adoptive transfer of stimulated Vγ9Vδ2 T cells *in vitro*, or by expanding them *in vivo* using suitable clinical grade agonists (253–259). In a study aimed at identifying molecular markers for susceptibility and resistance to Vγ9Vδ2 T cells in acute lymphoblastic leukemias and non-Hodgkin’s lymphomas, NRP2 was enriched in the resistant tumor samples (260). It is still unclear what exactly is the function of NRP2 in these cells and how it contributes to therapy resistance in the above settings. Given the fact that γδ T cells can be alternately polarized toward a protumor phenotype in the TME and NRP2 has been associated with immunosuppressive function in immune cells, it is possible that NRP2 expression in this T cell population results in their immunosuppression and immune evasion of the malignant cells. Hence, it is likely that NRP2 expression on the Vγ9Vδ2 T cells may emerge as a prognostic factor and target for immunotherapy. Recently, at the ATC, 2015, Nakayama et al. reported a novel immunomodulatory role of NRP2 in T cells. NRP2 was expressed in CD4^+^ effector T cells and Foxp3^+^CD4^+^ T_reg_ cells at baseline and was induced following exposure to mitogen such as CD3. This NRP2 then selectively binds to Sema3F and modulates T cell responses following transplantation. NRP2-depleted T cells were hyperproliferative and produced higher levels of IL-2, IL-17, and IFN-γ compared with wild-type cells. Also, mice with CD4^+^ T cell-specific deletion of NRP2 rejected minor MHC mismatched cardiac transplant faster than their wild-type counterparts, indicating hyperactivation of T cell response following transplantation. This is the first study, which documents an immunomodulatory role of NRP2 in T cells. Graft-versus-host disease (GVHD) is a common clinical complication that can arise following an allogenic transplantation and can be life threatening. Common treatment options include immunosuppressants. Based on this study, we can speculate that NRP2 may emerge as a lucrative therapeutic target for treating GVHD in the future.

The above findings document a myriad of functions for the NRPs in the development, activation, and function of different T cell subsets, in normal homeostasis as well as pathological conditions. Increasing number of reports has started to highlight the molecular pathways harnessed by NRP1 and NRP2 in T cells. It can be speculated that NRPs may emerge as potential targets for immunomodulating T cell response in various clinical disorders and open new vistas for treatment.

## NRPs in Other Immune Cell Compartments

Normal B cells do not express NRP1. However, NRP1 expression has been detected in B cells isolated from 7 out of 10 patients suffering from CLL (261). CLL B cells secrete VEGF and express VEGFR1 and VEGR2, which contribute to disease progression and resistance to apoptosis (262, 263). This suggests NRP1 as a coreceptor of VEGFRs, potentially plays an important role in CLL. However, larger cohort of patients needs to be studied to understand how NRP1 contributes to CLL progression. Till date, no studies have reported NRP2 expression in B cells, under normal physiological or clinical–pathological conditions.

Both NRP1 and NRP2 are expressed by human basophils and mast cells (264–266). Growing body of evidence suggests mast cells contribute to tumor progression and depletion of the former slows tumor growth (267, 268). Further studies are needed to elucidate the role of NRPs expressed on basophils and mast cells in tumor progression.

## Immunoregulation *Via* Manipulation of NRPs

It is now well appreciated that NRP1 and NRP2 have pleiotropic roles in different immune cell compartments in steady state as well as pathological conditions. They regulate a wide spectrum of functions, like migration, cell–cell interaction and immune response in myeloid and lymphoid cell subsets. Therefore, they can emerge as valuable therapeutic targets. Currently, we are not aware of any ongoing clinical trial targeting either NRP1 or NRP2 in any immune cell type in any pathological condition. Here, we will briefly discuss some of the potential therapeutic approaches to target the NRPs and develop effective immunotherapies in the future, especially for treating human malignancies.

Neuropilin-1 is often overexpressed in several malignancies and correlates with poor clinical outcome. Monoclonal antibodies (mAbs) targeting the three extracellular domains of NRP1 have been developed to block its coreceptor functions. For instance, mAb that specifically blocks VEGF165 binding to b1/b2 domain and thereby complex formation between VEGFR2 and NRP1, affected tumor-associated angiogenesis and tumor growth. MNRP1685A, another NRP1 mAb recently developed by Genentech, is in Phase I clinical trials, in combination with bevacizumab and or paclitaxel (269). Although in a recent Phase Ib study it was indicated that combination of MNRP1685A and bevacizumab cannot be used since it resulted in higher than expected proteinuria in patients for treating advanced solid tumors (270). One of the factors contributing to reduced therapeutic efficacy is that most of the drugs fail to penetrate the solid tumor beyond a depth of 3–5 mm, thus leaving most part of the tumor tissue viable. Tumor-penetrating peptides (TPP) may solve some of these limitations since they actively penetrate deep into the tumors and increase the efficacy of drug delivery, even without coupling of the drug to the peptide. The TPPs have motifs to bind to different receptors expressed on tumor endothelium or other cells in the TME but essentially contain a cryptic C-terminal motif (RXXR/K), called the CendR motif, essential for binding the b1/b2 domain of NRP1 (271–274). Many growth factors, including VEGF_165_, Sema3A, TGF-β use this motif to bind to NRPs (187, 275, 276). One prototypic example of TPP is iRGD, a cyclic peptide that contains a RGD motif for binding αv integrins predominantly expressed on tumor endothelium. Following initial binding with integrins, the iRGD peptide undergoes a proteolytic cleavage that exposes the CendR sequence that now binds NRP1 (and or NRP2) and gets internalized by a novel endocytic pathway activated by NRP1 (277). The unique endocytic vesicles formed by this pathway can accumulate a large volume of extracellular fluid and therefore any drug coadministered with the peptide will be able to access the tissue (bystander effect). TPPs can be used in combination with other mAbs or nanoparticles to increase their therapeutic efficacy (273, 278). sNRPs often act as decoys and antagonize NRP functions. Using sNRPs for therapy provides another alternative therapeutic approach. There is evidence that sNRP1 reduced tumor vasculature and tumor growth both in murine and human cells. Also, a mAb targeting NRP2 was developed. This blocks the binding of VEGFC to NRP2 without affecting Semaphorin binding and reduced tumor-associated lymphangiogenesis and metastasis (279). A mutant form of NRP2 (MutB-NRP2) that acts as a decoy and binds to VEGF with eightfold increased affinity compared with wild-type NRP2 reduced tumor burden in a xenograft model using melanoma cells, alone and when used in combination with bevacizumab (280, 281). Recently, Yang et al. isolated a mAb against NRP2 b1/b2 domain by hybridoma method (282). This antibody could bind full length NRP2 protein in cell lines. Extensive studies are required before this antibody can be used for therapeutic purposes. However, most of these studies have been performed in mouse model or *in vitro* using human cancer cell lines. It would be therefore important to study whether blocking NRP1 and/or NRP2 in human patients can be effective in targeting host immune system. As previously discussed immune regulation in tissue microenvironment in pathological conditions is often compromised. In tumors, for example, antitumor immune response is suppressed while the generation of immunosuppressive TAMs and Tregs is favored. In most cases, treatment of cancer patients with chemotherapy or radiotherapy, though initially reduces the tumor burden, eventually results in relapse of the disease with more aggressive features. In the initial phase, there is an increased infiltration of TAMs but most of the drugs induce increased production of M2 like cytokines and other factors, thus skewing these infiltrating macrophages more toward a protumorigenic phenotype. These then contribute to disease relapse. However, it should be noted that targeted therapies aimed at depleting TAMs or blocking their recruitment to TME have failed because macrophages are indispensable for eliciting antitumor immune response under favorable conditions. Since others and we have seen that NRPs do not affect macrophage recruitment in cancer, and that depletion of these two molecules can reeducate TAMs to activate antitumor immune response (Casazza et al. and unpublished data from our lab), targeting either molecule using blocking antibodies, for example, may prove beneficial for tumor patients in the future.

## Concluding Remarks

The function of NRPs in the immune system is an emerging field. In this review, we have attempted to summarize the current knowledge about the differential expression pattern and function of NRP1 and NRP2 in different immune cell compartments. Growing body of evidences indicate a requirement for both NRP1 and NRP2 in maintenance of immune homeostasis as well as their pathological contribution in various clinical disorders, and that they can be harnessed for developing effective immunotherapies in the future. However, there are many questions that remain unanswered. For instance, the precise role of NRPs in macrophages in normal physiology and pathological conditions, such as cancer, remains elusive. It is also not clearly understood why NRPs are upregulated by specific T cell subsets under defined conditions and the signaling pathways governed by these two molecules. Also, we do not know why they are expressed on other leukocytes like B cells and mast cells. We need to keep in mind that NRPs can exist as several splice variants that can differentially express under different conditions and exert complementary or opposite functions. For example, the two isoforms of NRP2, such as NRP2a and NRP2b, are endowed with opposite role in cancer progression. Till date, information about the differential expression pattern of the different NRP isoforms in the immune cell compartments is lacking. Designing immunotherapies such as cancer vaccines are likely to fail if we do not selectively target a specific isoform under a particular clinical setting. We believe that in the next few years, new reports focusing on the differential expression and function of different NRP isoforms will emerge. This will help us understand NRP biology and design effective immunotherapies in the future.

## Author Contributions

SR: planned and wrote the review article, as well as prepared the graphical representations. AB: planned and helped write the review and prepared the graphical representations in the manuscript. RS, JT, and SB: provided their expertise in writing the review article and also critically reviewed the manuscript. KD: helped in planning and critically reviewed the manuscript.

## Conflict of Interest Statement

The authors declare that the research was conducted in the absence of any commercial or financial relationships that could be construed as a potential conflict of interest.
